# Characterization of VldE (Spr1875), a Pneumococcal Two-State l,d-Endopeptidase with a Four-Zinc Cluster in the Active Site

**DOI:** 10.1021/acscatal.4c05090

**Published:** 2024-12-11

**Authors:** Vega Miguel-Ruano, Iván Acebrón, Mijoon Lee, Antonio J. Martín-Galiano, Celine Freton, Uxía P. de José, Balajee Ramachandran, Federico Gago, Morten Kjos, Dusan Hesek, Christophe Grangeasse, Leiv Sigve Håvarstein, Daniel Straume, Shahriar Mobashery, Juan A. Hermoso

**Affiliations:** † Department of Crystallography and Structural Biology, Consejo Superior de Investigaciones Científicas, Instituto de Química-Física “Blas Cabrera”, Madrid 28006, Spain; ‡ Department of Chemistry and Biochemistry, University of Notre Dame, Notre Dame, Indiana 46556, United States; § Core Scientific and Technical Units, Carlos III Health Institute, Majadahonda, Madrid 28222, Spain; ∥ Molecular Microbiology and Structural Biochemistry, CNRS UMR, Université de Lyon, Lyon 69367, France; ⊥ Department of Biomedical Sciences and IQM-CSIC Associate Unit, School of Medicine and Health Sciences, University of Alcalá, Alcalá de Henares 28805, Spain; # Department of Chemistry, Biotechnology and Food Science, Norwegian University of Life Sciences, Ås 1430, Norway

**Keywords:** VicRK regulon, pneumococcal stress response, cell-wall remodeling, l,d-endopeptidase, zinc-binding protein

## Abstract

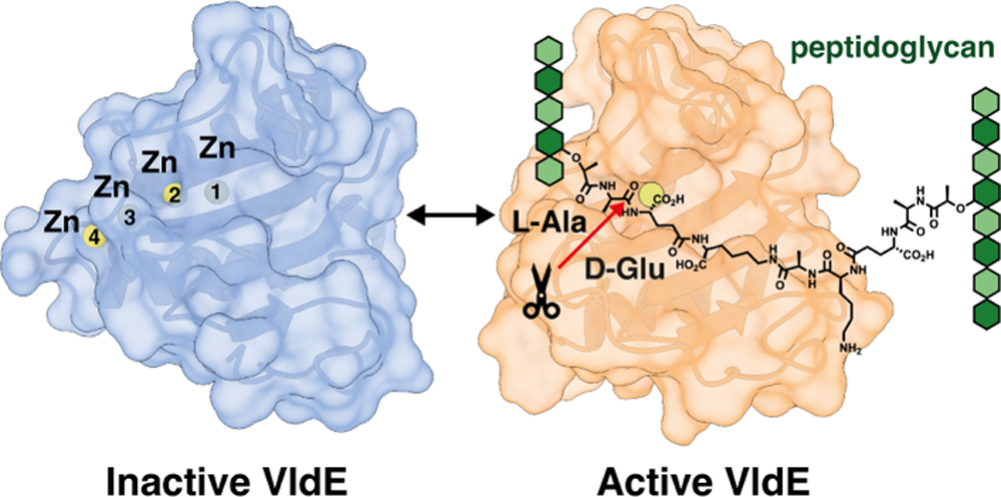

Remodeling of the pneumococcal cell wall, carried out by peptidoglycan (PG) hydrolases, is imperative for maintaining bacterial cell shape and ensuring survival, particularly during cell division or stress response. The *Streptococcus pneumoniae* protein Spr1875 plays a role in stress response, both regulated by the VicRK two-component system (analogous to the WalRK TCS found in Firmicutes). Modular Spr1875 presents a putative cell-wall binding module at the N-terminus and a catalytic C-terminal module (Spr1875^MT3^) connected by a long linker. Assays of the full-length protein and Spr1875^MT3^ with PG-based synthetic substrates by liquid chromatography/mass spectrometry revealed Spr1875 as an l,d-endopeptidase, renamed VldE (for VicRK-regulated l,d-endopeptidase), which hydrolyzed the cross-linked stem peptide in the PG. Remarkably, we observed asymmetric turnover with specific recognition of the acceptor peptide strand. Localization experiments showed that the protein is directed to the septum, which suggests that muralytic activity could be required for pneumococcal growth under stress conditions. Our findings, based on six high-resolution X-ray crystallographic structures and molecular-dynamics simulations, reveal two states for VldE^MT3^. The protein transitions between a noncatalytic state that binds up to four zinc ions, thus behaving as a Zn^2+^ reservoir, and a catalytic state that performs the hydrolytic reaction with a single zinc ion. Furthermore, computational studies provide insight into the mechanism of catalytic-water activation and nucleophilic attack on the specific scissile peptide bond of the asymmetric cross-linked PG.

## Introduction

**Figure 1:**
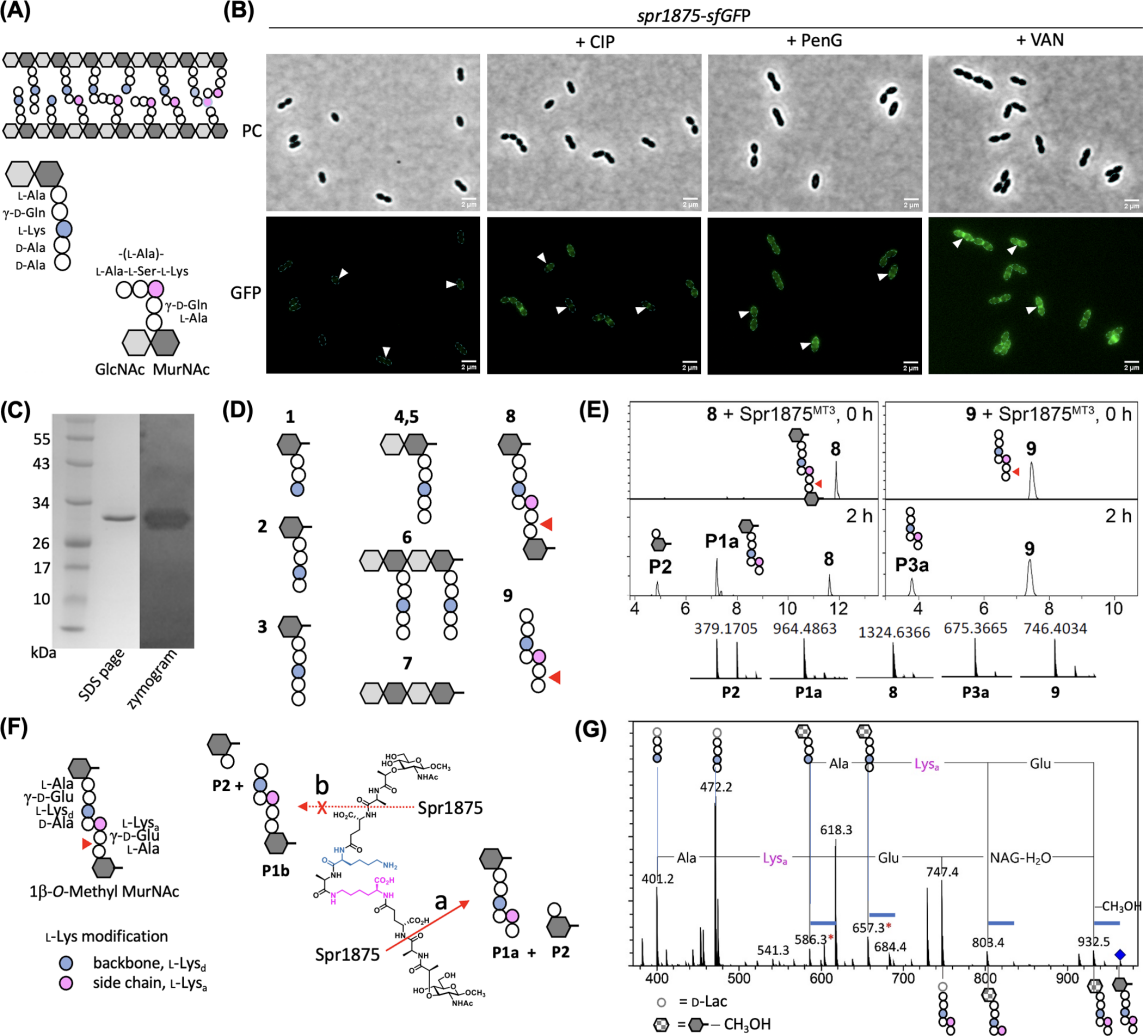
(A) Schematic peptidoglycan structure of *S. pneumoniae*. (B) Cells were grown in CY medium, then treated with 0.025 μg/mL Penicillin G (PenG) or 0.25 μg/mL Ciprofloxacin (CIP) or 0.25 μg/mL Vancomycin (VAN) for 1h at 37 °C and finally imaged on an agarose pad. Phase contrast (PC) (top row) and GFP fluorescent signal (bottom row) are shown for each condition. White arrowheads indicated division septa. Scale bar, 2 μm. (C) Muralytic activity of CHiC-Spr1875^MT3^. (D) Schematics of the structures of the synthetic PG fragments used for the assessment of substrate specificity of Spr1875, CHiC-Spr1875^MT3^, and Spr1875^MT3^ (**1**–**9**). (E) Reactions of Spr1875^MT3^ (VldE^MT3^) and compounds **8** (left panel) and **9** (right panel). The mass spectra of substrates and reaction products (bottom panel). The *m*/*z* values are given as [M + H]^+^. (F) Possible l,d-Endopeptidase reactions of Spr1875. (G) Collision-induced dissociation (CID) mass spectra of protonated reaction product P1a. The analysis of tandem mass spectra of the product P1a confirms that hydrolysis by VldE occurred at the bond indicated by the red arrow “a” based on the presence of fragment ions with 657 and 586.

The bacterial peptidoglycan (PG) is an essential protective barrier comprised of alternating units of *N*-acetylglucosamine and *N*-acetylmuramic acid, the latter decorated with a peptide stem. In *Streptococcus pneumoniae*, the composition of the pentapeptide stem is l-Ala-d(γ)-Gln-l-Lys-d-Ala-d-Ala (Figure 1A).^1^ The side chain of Lys can be modified with l-Ser-l-Ala or l-Ala-l-Ala. The bacterial cell wall is a dynamic structure, and its synthesis and remodeling proceed in concert during bacterial growth and division or during stress response, such as exposure to β-lactam antibiotics. PG hydrolases are important players in these cell-wall modifications. Bacterial response and adaptation to changes in the environment, such as the presence of antibiotics, is mediated by two-component system (TCS) signaling pathways, which trigger coordinated gene expression in response to the stress. In *S. pneumoniae*, the TCS VicRK, essential for bacterial growth, is also involved in cell-wall homeostasis as well as in virulence, biofilm formation, genetic competence, and fatty-acid metabolism.^2−6^ Certain PG hydrolases regulated by the VicRK regulon have been studied previously.^7−11^ The gene *spr1875* (as named in the *S. pneumoniae* strain R6, corresponding to spd1874 in strain D39) is part of the VicRK regulon and is conserved among pneumococcal serotypes.^12,13^*spr1875* is up-regulated (14–33-fold increase) in the absence of the essential lytic-cell-division protein PcsB,^14^ and it is highly overexpressed during antibiotic challenge.^15^ These findings suggest that it has a role in stress response and virulence.^12,16,17^ The protein encoded by the *spr1875* gene, Spr1875, is predicted to have a LysM cell-wall-binding domain (residues 47–92, per PROSITE),^18^ and a C-terminal MT3 (motif 3 present in the TM4 phage tape measure protein)^19^ putative hydrolytic domain (Spr1875^MT3^) (residues 267–379), as identified earlier,^19^ connected by a long, disordered linker (residues 93–266). In the present report, we show that this protein is directed to the septum and also that it has endopeptidase activity, which hydrolyzes the amide bond between l-Ala and d-Glu in the stem peptide. Furthermore, we demonstrate that Spr1875^MT3^ can alternate between two states: a noncatalytic state acting as a Zn^2+^ reservoir of up to four zinc cations and a catalytic state that organizes the catalytic machinery around a single zinc cation. Furthermore, a computational model was built that provides a detailed rationale for substrate recognition.

## Materials and Methods

### Construction of Bacterial Strains

Bacterial strains and plasmids used in the present study are described in Table S1. The oligonucleotides used for strain construction are listed in Table S2. To construct *S. pneumoniae* mutant strains gs551, ds1079, Spn 5, Spn 2211, Spn 2948, and Spn 2217, we used a two-step procedure based on a bicistronic *kan-rpsL* cassette called Janus,^20^ which allows a physiological level of expression of gene derivatives at their chromosomal locus.

### Bacterial Growth Conditions and Transformation

**Escherichia coli** was grown in lysogeny broth (LB) with shaking or on LB-agar at 37 °C. Plasmids were introduced into *E. coli* by heat shock, and transformants were selected on LB-agar containing a final concentration of 100 μg/mL ampicillin. *S. pneumoniae* was grown in C medium^21^ at 37 °C without shaking, or anerobically on Todd-Hewitt (TH) agar (Becton, Dickinson and Company) in an airtight vessel containing an AnaeroGen bag from Oxoid. Recombinant DNA was introduced into the genome of *S. pneumoniae* by inducing the bacteria to competence, as previously described by Hauge et al.^22^ Transformants were selected on TH-agar containing 400 or 200 μg/mL streptomycin.

### Phase Contrast and Fluorescence Microscopy

Strain ds353 and MK1206 (both expressing Spr1875-sfGFP from the native *spr1875* locus) were grown to early exponential-growth phase (OD_550_ = 0.1–0.2) and immobilized on a thin layer (<0.5 mm) of 1.2% (w/v) agarose in PBS covering a microscope slide. Microscopic images were taken using a Zeiss AxioObserver with ZEN Blue software, an ORCA-Flash 4.0 v2 Digital CMOS camera (Hamamatsu Photonics), and a 100x phase-contrast objective. An HXP 120 Illuminator (Zeiss) was used as a light source for fluorescence microscopy. Images were processed using ImageJ. Pneumococcal cells Spn 5, Spn 2211, Spn 2948, and Spn 2217 were grown in CY medium until OD_550_ = 0.1/0.2 and visualized using a Nikon TiE microscope fitted with an Orca-CMOS Flash4 V2 camera with a 100 Å ∼ 1.45 objective. For immunofluorescence microscopy, cells were mixed with either penicillin G at 0.025 μg/mL, ciprofloxacin at 0.25 μg/mL, or vancomycin at 0.25 μg/mL at 37 °C for 1 h and then imaged. Images were collected using the NIS-Elements (Nikon) and analyzed using the software ImageJ, GraphPad Prism, and the plugin MicrobeJ^23^ to generate fluorescent intensity and cell size violin plots.

### Pneumococcal Growth Experiments and Morphological Analyses

A 2-fold dilution series of penicillin G, ciprofloxacin (40–0 μg/mL), and vancomycin (20–0 μg/mL) were prepared in 150 μL volumes of C medium in a 96-well microtiter plate. The penicillin G concentrations ranged from 20 to 0 μg/mL for the R6 strain and from 80 to 0 μg/mL for the Pen6 strain. Exponentially growing bacteria (OD_550_ = 0.25–0.3) were diluted in fresh C medium to OD_550_ = 0.1, and 150 μL-aliquots of diluted cell culture were added to the wells in the 96-well microtiter plate, resulting in final OD_550_ = 0.05 and penicillin G concentrations from 10 to 0 and 40–0 μg/mL. The plate was incubated at 37 °C in a Synergy H1 Hybrid Reader (BioTek), and OD_550_ was measured automatically every 10 min for 20 h. For microscopic analyses, exponentially growing cells at OD_550_ = 0.1 were added to appropriate concentrations of penicillin G and grown for 2 h. Cultures without penicillin G were used as the controls. Phase contrast microscopy was performed as described above. The ImageJ software, GraphPad Prism, and the MicrobeJ plugin were used to examine cell morphology.^23^

### Cloning, Overexpression, and Purification of Spr1875 (VldE) and Spr1875^MT3^ (VldE^MT3^) Domain Variants (266–380 Construction)

To purify recombinant Spr1875 (without the signal sequence), we used a tandem affinity tag called CHiC (choline-binding histidine combination tag).^24^ The CHiC-encoding sequence was fused to the 5′ end of the *spr1875* gene starting at codon 24 using overlap extension PCR. All primers used in this article are listed in Table S2. The CHiC-encoding sequence was amplified from plasmid pGS01 using primers ds58 and ds317, and *spr1875* was amplified with primer pair ds163/ds165 using genomic DNA from strain RH425 as a template. The two PCR products were subsequently fused by performing overlap extension PCR using the primers ds317 and ds165. Similarly, the DNA sequence encoding the Spr1875^MT3^ domain was fused to the CHiC-encoding DNA fragment. The *spr1875*^*MT3*^ fragment was amplified from the genomic DNA of RH425 using primers ds165 and ds248 and subsequently fused to *CHiC* using primers ds317 and ds165. The *CHiC*-fused *spr1875* and *spr1875*^*MT3*^ fragments were cloned into pRSET A between the *Nde*I and *Eco*RI restriction sites to generate pRSET-CHiC-spr1875 and pRSET-CHiC-Mt3. The plasmids were transformed into *E. coli* DH5α, before they were isolated and transformed into *E. coli* BL21 (DE3). The plasmid pRSET-CHiC-Mt3 was used as a template to generate CHiC-fused point mutated versions of the Spr1875^MT3^ domain (H301A, D308A, H370A, D372A, H373A, H375A, and E368A). See Table S2 for a complete list of primers used to introduce point mutations and resulting plasmids.

*E. coli* BL21 (DE3) containing pRSET-CHiC-spr1875 or the different pRSET-CHiC-Mt3 variants was grown in LB medium containing ampicillin (100 μg/mL) to OD_600_ of 0.4, before protein production was induced by adding a final concentration of 0.1 mM isopropyl-β-d-thiogalactopyranoside (IPTG). The cultures were grown for another 4 h at 25 °C. CHiC-fused proteins were purified by DEAE-Sephacel (Sigma) affinity chromatography, as previously described by Stamsås et al.^24^ The volume of purified fusion proteins was reduced to 1 mL using Amicon Ultra filters (10,000 MW) and dialyzed against 10 mM Tris-HCl, 150 mM NaCl, 140 mM choline-chloride at room temperature for 1 h to remove excess NaCl. The CHiC-tag was cleaved off of CHiC-fusions by adding a final concentration of 1 mM DTT and 10 μg/mL of 6xHis-TEV protease. The cleavage reaction was performed at room temperature overnight. Then the samples were diluted 10-fold in 10 mM Tris-HCl and 150 mM NaCl. The noncleaved fusions and free CHiC-tag were removed from the target protein by performing a second DEAE-Sephacel chromatography (Ni^2+^ IMAC, which is usually performed to remove His-TEV and CHiC-tag, was not applicable due to the multiple histidine residues in the catalytic cleft of the Spr1875^MT3^ domain). The volume of the DEAE-Sephacel flow-through (containing the target protein) was reduced to 0.5 mL using Amicon Ultra filters (10,000 MW). Traces of His-TEV, cleaved CHiC-tag, and noncleaved CHiC-fusions were finally removed by size-exclusion chromatography on a Superdex 75 10/300 GL column equilibrated in 10 mM Tris-HCl, 150 mM NaCl using a flow rate of 0.3 mL/min.

### SDS-PAGE and Zymography Experiments

SDS-PAGE was performed as described by Laemmli,^25^ and zymography was performed according to the protocol of Eldholm et al.^26^ Briefly, 2.5 μg of each purified protein were separated in a 12% polyacrylamide resolving gel for Coomassie blue staining or in a 12% resolving gel containing heat-inactivated (95 °C for 10 min) RH14 cells from a 300 mL cell culture at OD_550_ = 0.2 for zymography. A 4% stacking gel was used in both cases. After electrophoresis, the zymogram was washed 2 × 30 min in dH_2_O with gentle shaking before the gel was submerged in 25–30 mL refolding buffer (50 mM NaCl, 20 mM MgCl_2_, 0.5% Triton X-100, and 20 mM Tris-HCl, pH 7.4). The zymogram was developed overnight at room temperature with gentle shaking.

### Synthesis of Peptidoglycans **1**–**7**

Peptodiglycans **1**–**7** were prepared by literature procedures.^27−30^

#### Synthesis of Endopeptidase Substrates **8** and **9**

**Scheme 1:**
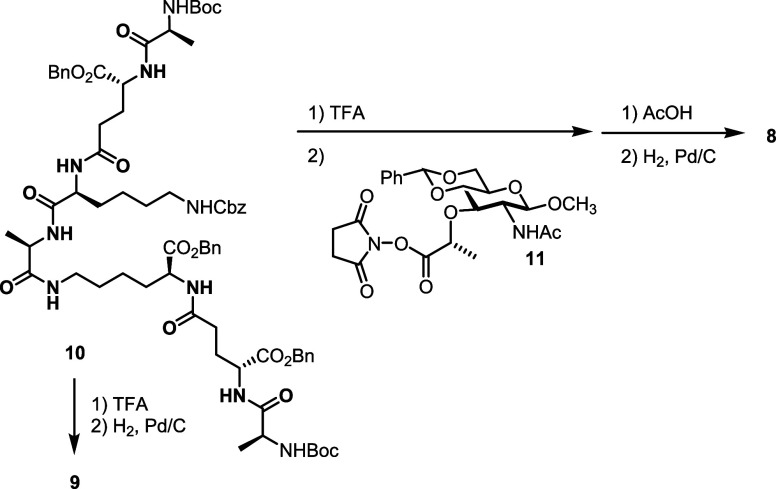
Synthesis of Endopeptidase Substrates **8** and **9**

The synthesis of endopeptidase substrates **8** and **9** is not reported previously and is outlined in Scheme 1. The coupling reaction of the protected Lys-heptapeptide, **10**(30) with activated ester **11** and subsequent deprotection to give **8** and the direct deprotection of **10** to give **11** were done using the general methods reported previously.^30^

##### Compound **8**

^1^H NMR (500 MHz, D_2_O) δ 1.34 (d, *J* = 6.8 Hz, 6H), 1.40 (d, *J* = 7.1 Hz, 3H), 1.37–1.83 (m, 12H), 1.94 (s, 6H), 1.97–2.38 (m, 8H), 2.96 (t, *J* = 7.3 Hz, 2H), 3.11–3.21 (m, 2H), 3.41–3.52 (m, 6H), 3.46 (s, 6H), 3.48–3.52 (m, 6H), 3.72–3.92 (m, 6H), 4.16–4.38 (m, 11H); ^13^C NMR (126 MHz, D_2_O) δ 16.71, 16.95, 18.73, 22.21, 22.33, 22.41, 26.47, 26.64, 26.77, 27.93, 30.13, 30.30, 31.34, 31.51, 39.03, 39.29, 49.66, 49.72, 50.12, 51.98, 52.09, 52.95, 54.29, 55.03, 55.05, 57.26, 60.82, 68.95, 69.00, 75.76, 78.24, 82.63, 82.68, 102.05, 174.24, 174.32, 174.34, 174.58, 174.70, 174.93, 174.98, 175.02, 175.18, 175.49, 175.55, 175.90; HRMS (ESI), calcd for C_55_H_93_N_11_O_26_ (M+H+), 1324.6366, found 1324.6380.

##### Compound **9**

^1^H NMR (500 MHz, D_2_O) δ 1.40 (d, *J* = 7.1 Hz, 3H), 1.58 (2d, *J* = 7.1 Hz, 6H), 1.35–1.89 (m, 12H), 2.03–2.48 (m, 8H), 3.01 (t, *J* = 7.6 Hz, 2H), 3.18–3.27 (m, 2H), 4.16 (dq, *J* = 1.9, 7.1 Hz, 2H), 4.23–4.28 (m, 2H), 4.33 (dd, *J* = 5.1, 8.8, 7.1 Hz, 1H), 4.42 (dt, *J* = 5.1, 14.2 Hz, 2H); ^13^C NMR (126 MHz, D_2_O) δ 16.73, 16.76, 22.23, 22.43, 26.49, 26.56, 26.68, 27.95, 30.17, 30.38, 31.34, 31.45, 39.06, 39.30, 49.22, 50.12, 52.42, 52.45, 52.99, 54.19, 170.99, 171.02, 174.24, 174.76, 174.86, 174.94, 175.02, 175.13, 176.08; HRMS (ESI), calcd for C_31_H_55_N_9_O_12_ (M+H^+^), 746.4034, found 746.40430.

### Enzyme Activity Screening Assay Using UPLC/MS and UPLC/MS/MS

Reactions of each enzyme construct (Spr1875^MT3^, CHiC-1875^MT3^, and Spr1875) with each peptidoglycan substrates (**1**–**9**) were carried out in 10 mM Tris-HCl, pH 7.4, 150 mM NaCl at room temperature. At each time point, sample aliquots were taken, heated for 5 min at 100 °C, and flash frozen until UPLC/MS. UPLC/MS and UPLC/MS/MS analyses were done by the method reported previously^31^ with slight modification of LC gradients. Briefly, the UPLC/MS instrument consisted of a Waters Acquity H-Class UPLC equipped with a Waters Acquity sample manager-FTN and PDA detector coupled with a Bruker impact II ultrahigh resolution Qq-time-of-flight hybrid mass spectrometer controlled by a Bruker Compass HyStar version 5.0 SR1. The Bruker electrospray ionization source was operated in the positive ion mode with the following parameters: end plate offset voltage = −500 V, capillary voltage = 1800 V, nitrogen as both a nebulizer (4 bar) and dry gas (7 L/min) at 200 °C, funnel 1 RF = 300 V, funnel 2 RF = 400 V, hexapole RF = 300 V, quadrupole ion energy = 5 V, collision energy = 5 eV, collision cell RF = 2500 V, ion transfer time = 120 μs, and prepulse ion storage = 10 μs. Mass spectra were accumulated over the mass range 200–3000 *m*/*z*. A 20 min LC gradient was used for analysis of **1**–**8**: 2% B for 2 min, 2% B to 15% B for 13.9 min, 15% B to 2% B for 0.1 min, and 2% B for 4 min (where A = 0.1% formic acid in water, B = 0.1% formic acid in acetonitrile). A 15 min LC gradient was used for the analysis of **9**: 0% B for 5 min, 0% B to 15% B for 5.9 min, 15% B to 0% B for 0.1 min, and 0% B for 4 min. The LC flow for the first 3 min was diverted to waste. UPLC/MSMS was performed in Auto MS/MS mode (Bruker otofControl 5.2) using collision-induced dissociation (CID) with argon as the collision gas to confirm the chemical structures of reaction products P1a and P3a. The specific collision energies used for P1a (*m*/*z* = 964.49) and P3a (*m*/*z* = 675.37) were 48.9 and 40.3 eV, respectively, and their CID spectra are shown in Figures 1D and S7B.

### Intact LC/MS Analysis of Three Enzyme Constructs

A 15 min LC gradient (10% B for 2 min, 10% B to 90% B for 10.9 min, 90% B to 10% B for 0.1 min, 10% B for 2 min) with an Agilent Poroshell 300SB-C3 column (5 μm, 2.1 × 75 mm) was used. The UHPLC/MS instrument consisted of a Dionex Ultimate 3000 Rapid Separation system equipped with a Dionex Ultimate 3000 autosampler and a Dionex Ultimate 3000 photodiode array detector coupled to a Bruker MicrOTOF-Q II quadrupole time-of-flight hybrid mass spectrometer using Hystar 3.2 SR4 software. The Bruker electrospray ionization source was operated in the positive ion mode with the following parameters: end plate offset voltage = −500 V, capillary voltage = 1800 V, nitrogen as both a nebulizer (4 bar) and dry gas (8 L/min) at 180 °C, funnel 1 RF = 400 V, funnel 2 RF = 400 V, hexapole RF = 400 V, quadrupole ion energy = 6 V, collision energy = 6 eV, collision cell RF = 1200 V, ion transfer time = 130 μs, and prepulse ion storage = 25 μs. Mass spectra were accumulated over the mass range 400–3000 *m*/*z*. Multiply charge ions were deconvoluted using the maximum entropy algorithm (Compass DataAnalysis), and deconvoluted mass spectra are given in Figure S5.

### Nano-Differential-Scanning Fluorimetry (nanoDSF)^32,33^

The analysis was done in Tycho NT.6 equipment, measuring the intrinsic protein fluorescence (tryptophan and tyrosine) at 330 and 350 nm over a temperature range of 35–95 °C. The inflection points in the curve represent melting temperatures, temperatures at which structural transitions occur. VldE^MT3^ denaturation profiles were also evaluated in the presence of 20 μM of ZnCl_2_ (VldE^MT3^ + ZnCl_2_) and in the presence of 20 μM of EDTA (VldE^MT3^ + EDTA). Protein concentration was adjusted to 5 μM in 20 mM Tris pH 7.4 and 150 mM NaCl, and 10 μL of each sample were introduced in a thin capillary. Each condition was subjected to replicates (*n* = 5 for the VldE^MT3^, *n* = 4 for the VldE^MT3^ + ZnCl_2_, and *n* = 3 for the VldE^MT3^ + EDTA).

### Sedimentation-Velocity Analytical Ultracentrifugation

The protein VldE^MT3^ was suspended in a buffer containing 20 mM Tris-HCl pH 7.4, 150 mM NaCl at 0.05 μM corresponding to OD_600_ values of 0.76. Sedimentation-velocity experiments were performed in an XLA Beckman-Coulter ultracentrifuge at 48 krpm and 20 °C. Absorbance at 280 nm was measured, and specific volume, buffer density, and viscosity were calculated. The *s*_20,w_ value of the peak at 1.797 S (100%) corresponds to the molecular weight for the monomeric protein. Data analysis was performed in the SEDFIT 16.36 software package using a 0.68 confidence level (*F*-ratio).

### Crystallization of VldE^MT3^ Zinc-Binding Structures

VldE^MT3^ protein was concentrated up to 33–70 mg/mL using an Amicon Ultra-4 Centrifugal Concentrator for a 3 kDa MWCO cutoff from Millipore. High-throughput crystallization trials were performed in 96-well plates at 18 °C, using commercial conditions from Hampton Research, Jena Bioscience, Qiagen and Molecular Dimensions, and an Innovadine crystallization Robot. Sitting drops consisted of 250 nL of the protein and 250 nL of the reservoir condition in vapor equilibration with 65 μL of reservoir. Conditions that produced crystals were optimized and scaled up to 1 μL of protein, 1 μL of the crystallization condition, and 150 μL of reservoir. The initial VldE^MT3^ structure, VldE^MT3^:4Zn structure, was obtained in 100 mM Hepes at pH 7.5, 50 mM cadmium sulfate, and 1 M sodium acetate at 70 mg/mL. A total of 20% of glycerol was used as a cryoprotectant. VldE^MT3^:3Zn and VldE^MT3^:1Zn structures were obtained in the same crystallization condition, supplementing the protein (52 mg/mL) and the crystallographic reservoir with 5 mM of EDTA. VldE^MT3^:2Zn structure was obtained at 33 mg/mL by varying the pH of the crystallization condition, 100 mM Tris-HCl pH 8.5, 50 mM cadmium sulfate, and 1 M sodium acetate.

### Crystallization of VldE^MT3^|Act Structure

VldE^MT3^ sample, at 50 mg/mL, was incubated with 5 mM EDTA. Crystallization experiments were conducted in 100 mM Hepes pH 7.5, 50 mM cadmium sulfate, and 1 M sodium acetate. The resulting crystals were fast-soaked with a higher concentration of EDTA (20 mM), yielding the VldE^MT3^|act structure at 1.5 Å resolution. A total of 20% of glycerol was used as a cryoprotectant.

### Production and Crystallization of the Inactive Versions of VldE^MT3^

To explore the structural features underlying the VldE substrate preference, we generated catalytically impaired mutants for crystallographic experiments with the synthetic substrate (**9**). This involved the production of single (H373A), double (H370A and H373A), and triple (H370A, H373A, and E368A) mutants in the L_zz_ region. Double- and triple-altered versions of the protein displayed limited solubility and diminished structural stability, as determined by the melting temperature by nanoDSF. Attempts to crystallize these versions failed. The H373A variant, however, led to success in crystallization. VldE^MT3^ H373A sample was dialyzed before the crystallographic experiments to remove traces of choline from the purification, using 10 mM Tris HCl pH 7.0 and 150 mM NaCl. The sample was concentrated up to 66 mg/mL using an Amicon Ultra-4 Centrifugal Concentrator, 3 kDa MWCO cutoff from Millipore. The best crystals were obtained after 5 days in 100 mM Hepes at pH 7.2, 50 mM cadmium sulfate, and 0.6 M sodium acetate.

### Data Collection and Structural Determination

Diffraction data sets were collected at the ALBA Synchrotron using the XALOC beamline and a Pilatus 6 M detector. Crystals from the zinc-binding structures (VldE^MT3^:4Zn, VldE^MT3^:3Zn, VldE^MT3^:2Zn, and VldE^MT3^:1Zn) were collected at the K-edge maximum absorption of Zn, 1.283 Å. Alternatively, crystals for the VldE^MT3^|act and VldE^MT3^H373A inactive versions were collected at 0.979 Å. However, additional crystals for these conformations were measured at 1.283 Å to confirm the presence of Zn-1, although they are not included in Table S3. All data were reduced using XDS^34,35^ and scaled using Aimless^36^ from the CCP4 program suite.^37^ The CC1/2 > 0.3 criterion was used to determine the diffraction-limit surface for data. Phase solving was initially performed by the ARCIMBOLDO ab initio method,^38^ based on locating small model fragments such as α-helices. Models were then manually completed using Coot^39^ and refined using Phenix^40,41^ and Refmac5.^42^ For the VldE^MT3^:3Zn, VldE^MT3^:1Zn, and VldE^MT3^ H373A structures, twinning was detected, and the *–h, −k, l* twin law was applied for refinement. All the structures were visualized with PyMol.^43^

All of the structures belong to the P3 space group, with one molecule in the asymmetric unit and 43–51% of solvent, and dimensions are shown in Table S3. Geometry and bond distances are characteristic of zinc coordination with the following bond lengths: Zn-Asp between 2.0 and 2.2 Å, Zn-Glu between 2.0 and 2.3 Å, Zn-His between 1.9 and 2.5 Å, and Zn-Water between 1.9 and 2.4 Å.^44^ Cadmium and sulfate ions from the crystallographic condition were also observed in the electron density involved in crystal packing. A summary of the crystallographic data is given in Table S3.

### Molecular Dynamics (MD) Simulations

The L_zz_ closed conformation observed in the VldE^MT3^:1Zn crystallographic structure was subjected to MD simulations to explore its conformational stability over time (Movies S1 and S2). Additionally, MD simulations were conducted to model the substrate recognition by VldE^MT3^. Based on the structural comparison of VldE^MT3^ protein with the related M15 and M23 enzymes, we inferred that (i) N^e^ of H373 donates a hydrogen bond to the TS water oxygen as observed for W166 in VanXYg (PDB id. 4MUQ)^45^ when complexed to a phosphinate transition-state analog for the hydrolysis of the d-Ala-d-Ala amide, and (ii) the peptide carbonyl oxygen is held in place by both the Zn^2+^ ion and the guanidinium of R294. Simulation of a minimalist transition-state species for the reaction—consisting of a tetrahedral species in place of the amide in the l-Ala-d(γ)-Glu moiety bound to VldE^MT3^—allowed us to support this proposal (Movie S3). We subsequently built the structure into larger PG fragments **8** and **9** that we had shown experimentally to be substrates for the enzyme (Movie S4).

MD simulations were carried out using the *pmemd.cuda_SPFP* module of AMBER 18^46^ and the ff14SB force field for protein atoms. For the metal centers, the bonded plus electrostatics model implemented in ZAFF and EZAFF^47,48^ was employed, following the assignment of ZN9, HD8, HE5, and AP2 residue types to Zn^2+^, H301, H375, and D308, respectively. Nonstandard residues for the substrates and transition states were built in a manner consistent with the AMBER force field using atom-centered RESP charges,^49^ derived using a 6-31G* basis set, the density functional tight-binding method, and the IEF-SCRF continuum solvent model^50^ for water, as implemented in the Gaussian program.^51^ Each modeled system was immersed in a cubic box of TIP3P water and neutralized by the addition of counterions. Electrostatic interactions were represented using the smooth particle-mesh Ewald method with a grid spacing of 1 Å and a cutoff distance of 9 Å for the nonbonded interactions. Energy minimization was carried out by performing 5000 steps of steepest descent, followed by 50,000 steps of conjugate-gradient energy minimization. Then, the system was heated up to 300 K applying a restraint of 0.5 kcal mol^–1^ Å^–2^ on backbone atoms and relaxed before the MD production through an equilibration phase. MD runs of at least 300 ns were then launched by using the equilibrated system as a starting point. The VMD^52^ and PyMOL molecular-graphics programs were used for visualization of the MD results and movie generation.

## Results

### Spr1875 Contributes to the Growth of a Penicillin-Resistant Strain in the Presence of Antibiotics and Localizes to the Septum

Upregulation of *spr1875* by exposure to penicillin was confirmed by measuring fluorescence intensity using a *sfGFP*-fused construction in the R6 strain (see Methods and Table S1 for a complete list of strains). An increase of signal was detected after exposure of bacteria to 0.025 μg/mL of penicillin G (PenG) within one hour (Figure S1). Surprisingly, we made a similar observation upon exposure to 0.25 μg/mL vancomycin or ciprofloxacin (Figure S1), indicating that *spr1875* expression is triggered upon different antibiotic-induced stress. The functionality of Spr1875-sfGFP was checked by comparing the cell area of WT-R6 and *spr1875-sfGFP* cells grown in the presence of antibiotics (Figure S2). This fluorescent construct was also used for localization, which showed that Spr1875 is mainly localized at the cell center (division septum) irrespective of the antibiotic utilized and the cell cycle stage. (Figures 1B and S3), This suggested that *spr1875* may have a role in remodeling newly synthesized cell wall. We also assessed the impact of deleting *spr1875* on pneumococcal cell growth and cell morphology in different strains and/or the presence of antibiotics (Figures S4 and S5).

Remarkably, deletion of *spr1875* in a nonpenicillin-resistant strain (R6) did not strongly impact cell growth,^16^ but Δ*spr1875* cells exposed to 0.075 μg/mL PenG were somewhat larger than wild type (Figure S4A). Conversely, in the resistant-strain Pen6, deletion of *spr1875* significantly affected cell growth and morphology and delayed the onset of autolysis upon PenG exposure (Figure S4B). By contrast, we did not detect any cell growth and shape defects between WT-R6 and Δ1875-R6 strains when stressed with vancomycin or ciprofloxacin (Figure S5). These findings indicated that cells expressing Spr1875 are better suited to handle penicillin-induced stress.

### Spr1875 Is a Peptidoglycan l,d-Endopeptidase

To investigate the putative cell-wall lytic role of Spr1875, we performed zymography using purified Spr1875^MT3^ fused to a choline-binding histidine combination tag (CHiC) (see Tables S1 and S2 for lists of plasmids used for protein overexpression and DNA oligos for genetic constructs).^24^ Clearance of pneumococcal crude sacculi shows that Spr1875^MT3^ is capable of hydrolyzing the PG (Figures 1C and S6). In order to identify the potential amino acids responsible for this activity, we expressed Spr1875^MT3^ variants mutated in positions conserved in other proteins related to the tape-measure protein TM4MT3^19^ and in other metallopeptidase catalytic domains.^53^ Amino acids H301, D308, and H375 were predicted to be involved in metal coordination.^53^ The muralytic activity was lost when we performed alanine substitutions at these positions (Figure S6). Also, substituting residues H370, D372, and H373 individually for alanines (highly conserved in proteins containing the MT3 motif)^19^ rendered the enzyme inactive, indicating potential roles for these residues in the catalytic mechanism (Figure S6). Conversely, the E368A variant, described later in the article, retained the activity (Figure S6).

To elucidate the nature of the specific substrate for the Spr1875^MT3^ domain, we performed assays using several synthetic peptidoglycan fragments (Figures 1D and S7). It is worth mentioning that most of our synthetic PG fragments contained the nonamidated version of the peptide at position 2 (i.e., Glu instead of Gln). Three different constructs of Spr1875 were evaluated: the full-length protein, the CHiC-fused Spr1875^MT3^ domain, and the Spr1875^MT3^ alone. The three constructs were checked by LC/MS, and the deconvoluted masses agreed with the theoretical values within 1 Da (Figure S8). We screened the activities of the three constructs with nine synthetic PG fragments (Figures 1D and S7). Full chemical structures of peptidoglycan substrates are given in Figure S7. These samples were prepared in multistep syntheses, per methods described earlier,^27−30^ and cross-linked peptides **8** and **9** were synthesized for these studies (Scheme 1). d,d-, l,d-Carboxypeptidases, d,d-, l,d-endopeptidases, l-alanine amidase, and lytic transglycosylase activities were explored. Among them, all three constructs showed l,d-endopeptidases activity on the cross-linked asymmetric substrates **8** and **9** (Figures 1E and S9), and the reactions of Spr1875^MT3^ are shown as representative examples in Figure 1E. After the addition of the enzyme to **8**, two products formed (*t*_R_ = 5 and 7 min), while the amount of substrate **8** (*t*_R_ = 11.5 min) decreased. The MS analysis of the two products (P2 and P1a; Figure 1E) showed *m*/*z* of 379 and 964 Da, respectively. This indicated that the hydrolyzed bond is between l-Ala and d-Glu (red triangles and red arrow in Figure 1). Accordingly, we hereby rename Spr1875 as VldE (for VicRK-regulated l,d-endopeptidase). Remarkably, as compounds **8** and **9** are indeed substrates for VldE, our results demonstrate that amidation of the Glu residue is not essential for the enzyme’s activity.

Interestingly, there are two hydrolyzable bonds in substrate **8** and thus, two possible hydrolytic routes (designated as *a* and *b* in Figure 1F). Turnover of **8** by VldE could produce a mixture of P1a and P2, or it could go selectively to P1b and P2. Masses of potential products P1a and P1b are identical, and these two products can only be differentiated by MS/MS analysis. Indeed, the analysis of the fragmentation pattern by tandem mass spectrum of product P1a concluded that hydrolysis occurs exclusively via route *a* (Figures 1F,G and S10A). The same selective reaction outcome was observed with substrate **9** (Figure S10B). The chemical structures of products P1a and P3a indicated that hydrolysis catalyzed by VldE occurred only at the bond in an acceptor strand of the cross-linked peptide substrates. This explains why l,d-endopeptidase activity was not detected with PG fragments 1–6 with non-cross-linked peptides. These compounds contain the l,d-bond between l-Ala and d-Glu at the donor strand.^16^

### Crystal Structure of VldE^MT3^ with an In-Line Four-Zinc Binding Site

**Figure 2:**
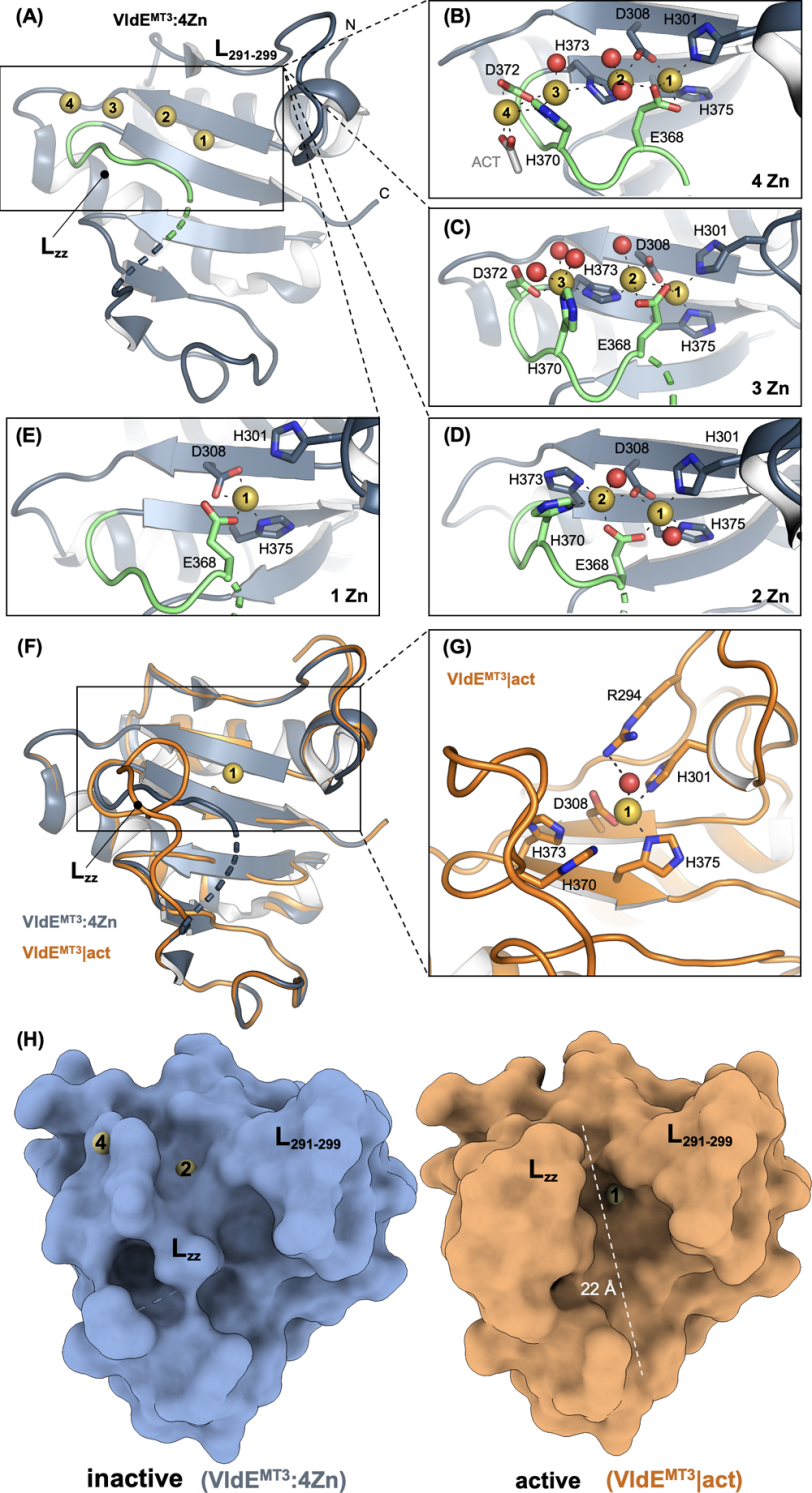
(A) Overall crystallographic structure of the VldE^MT3^ domain with a four-zinc binding site, VldE^MT3^:4Zn, represented in blue cartoon. L_zz_ is highlighted in green. Zinc ions are shown as yellow spheres, numbered 1–4. (B–E) Detailed view of the zinc-binding cleft in the four closed structures containing four (VldE^MT3^:4Zn), three (VldE^MT3^:3Zn), two (VldE^MT3^:2Zn), and one (VldE^MT3^:1Zn) zinc ions, respectively. Residues coordinating zinc ions are shown as sticks. Water molecules are shown as red spheres. (F) Comparative structure overlay of the VldE^MT3^ zinc binding (VldE^MT3^:4Zn) and catalytically competent (VldE^MT3^|act) structures, depicted in blue and orange, respectively. (G) Details of the catalytic machinery of VldE^MT3^ are shown. Key residues are represented as orange sticks. (H) Surface view comparison of VldE^MT3^|act, in orange, and VldE^MT3^:4Zn, in blue, revealing an expansive cavity of VldE^MT3^|act defined by L_291–299_ and L_zz_ regions.

The VldE^MT3^ domain, residues 266–380, was expressed and purified. Sedimentation-velocity-ultracentrifugation analysis indicated that VldE^MT3^ domain exist as a monomer in solution (Figure S11). Crystallization experiments at pH 7.5 (see Methods) allowed the structure determination of VldE^MT3^ at 1.5-Å resolution (Figure 2A). This structure presents an α/β arrangement formed by four α-helices (α1-α4) and six antiparallel β-strands (β1-β6) (Figure S12), a fold shared by the LAS family (Lysostaphin-type enzymes, d-Ala-d-Ala metalloproteinases and the zinc-ion-dependent peptidase of the sonic hedgehog protein).^54^ Unexpectedly, four Zn^2+^ cations were found bound in tandem to the cleft region (VldE^MT3^:4Zn complex, Figure 2A) despite the fact that no exogenous zinc ion was added during the crystallization steps. This finding underscores the high affinity of VldE for binding multiple zinc ions simultaneously. An X-ray fluorescence scan confirmed the presence of these cations, revealing the characteristic scattering factors for zinc (Figure S13). Crystallographic data, collected at the K-edge maximum absorption of Zn (1.283 Å), provided anomalous difference maps at each of the four zinc-ion positions (Figure S13A). This is, to our knowledge, the first case of a metalloprotease containing more than two zinc ions at the active site.

Structural homologues to VldE^MT3^, according to DALI,^55^ include d,d-carboxypeptidases and endolysins from bacteriophages, such as the *Streptococcus albus* DCC metallopeptidase (PDB: 1LBU),^56^ the *Bacillus cereus* bacteriophage B4 LysB4 endolysin (PDB: 6AKV),^57^ and the *Escherichia* phage T5 EndoT5 l-Ala,d-Glu peptidase (PDB: 2MXZ)^58^ (Figure S14). All of these homologues exhibit a single catalytic zinc cation (Figure S14). We assign Zn-1 in VldE^MT3^ to the conserved catalytic zinc ion seen in all metallopeptidases. Zn-1 is coordinated by H301, D308, and H375 (Figure 2B), thus revealing that VldE^MT3^ belongs to the Clan MD of metallopeptidases, a group that includes peptidases involved in bacterial cell-wall biosynthesis and lysis.^59^ The stabilization of the additional zinc ions (Zn 2–4) in VldE^MT3^ is facilitated by a flexible loop (amino acids 360–372), which we annotate here as the zinc-zipper loop, L_zz_ (Figure 2A,B). L_zz_ presents a conformation that closes the binding cleft with the E368 residue coordinating Zn-1. The canonical HxH motif found in LAS metallopeptidases (H373-X-H375)^53,59,60^ is hereby expanded by two acidic residues, D372 and E368, and another histidine (H370), both from the L_*zz*_ (Figure 2B). Structural comparison (Figure S14) reveals that VldE^MT3^ exhibits significant differences in L_*zz*_ and L_291–299_ by altering the catalytic cleft (Figure S14).

Nanodifferential scanning fluorimetry (nanoDSF) was employed to evaluate the affinity of VldE^MT3^ protein for zinc ions in solution. Analysis of the thermal unfolding revealed that the removal of Zn^2+^ by EDTA destabilizes the protein, while ZnCl_2_ supplementation had a stabilizing effect (Figure S15).

### L_zz_ Region Is Essential in Modulating VldE^MT3^ Zinc-Binding Plasticity

To investigate the role of L_zz_ in modulating the VldE^MT3^ zinc-binding ability, we performed crystallographic experiments to remove Zn^2+^ by adding 5 mM EDTA to the protein. These experiments resulted in two new crystal structures; one with 3 zinc ions in the binding cleft (VldE^MT3^:3Zn, Figure 2C) solved at 1.6 Å resolution and another with a single zinc ion (VldE^MT3^:1Zn, Figure 2E) solved at 2.8 Å resolution. Another crystal structure with two zinc ions solved at 1.85 Å resolution (VldE^MT3^:2Zn, Figure 2D) was also obtained by varying the pH of the crystallization experiment (pH 8.5). Crystallographic experiments with EDTA concentrations as high as 20 mM failed to remove Zn-1 from the active site. We take this as an indication that this Zn^2+^ is a key element of the structure of VldE, which is also consistent with the fact that stabilization of Zn-1 in all four crystallographic structures involved the participation of a greater number of amino acids, in contrast to Zn-2, Zn-3, and Zn-4 (Figures 2 and S16A).

The four structures present a similar overall closed conformation for the active site, showing small differences in the arrangement of loops L_291–299_ and L_310–315_ for VldE^MT3^:1Zn (Figure S16B). The L_zz_ loop exhibits strong plasticity in the four structures (Figure S16) with residues H370, E368, and H373 reorienting their side chains to stabilize variable numbers of Zn^2+^ cations (Figure 2B–E). Analysis of these four structures confirms the dynamic nature of the L_zz_ region, as only residues involved in the interaction with Zn^2+^ (amino acids 368–372) are visible in the electron-density maps, while the remaining ones (amino acids 363–367) are not (Figure S16C). None of the four different structures are compatible with an active catalyst as the L_zz_ region, and particularly residue E368, blocks access to Zn-1. Independent results from MD simulations on the VldE^MT3^:1Zn closed structure (Movie S1) confirmed the stability of this closed conformation.

### Three-Dimensional Structure of a Catalytically Competent Conformation of VldE^MT3^

A new crystal structure showing a catalytically competent arrangement (VldE^MT3^|_act_) was obtained at increasing concentrations of EDTA (see Methods) and solved at 1.5 Å resolution (Figure 2F,G). The fold is similar to that of other active metallopeptidases reported earlier (Figure S17)^56−58,61−66^ and to the AlphaFold prediction (UniProt ID: Q8DN78) (Figure S18). In this new conformation, the L_zz_ region undergoes refolding that promotes the outward displacement of E368 from the active site and allows the arrangement of catalytically competent machinery around Zn-1 (Figure 2G). A single zinc ion, Zn-1, was found fully occupying the cleft, while a residual occupancy (0.5) for a noncatalytic Zn-3 was also observed. Loops, L_zz_ and L_291–299_, now define a large groove of 22 Å (Figure 2H). The catalytic Zn-1 is coordinated by H301, D308, and H375 and also by a water molecule, which is stabilized by R294 from one of the cavity-defining loops, L_291–299_ (Figure 2G). This arginine, together with R363 of L_zz_, frames the catalytic cleft.

The three-dimensional structure of the H373A variant solved at 1.14 Å resolution (Table S3) confirmed a conformation similar to the VldE^MT3^|act (Figure S18C). Attempts to incubate the protein or crystals with synthetic compound **9** failed to yield a structure for the complex. Computational experiments were thus employed to model the complex.

### Molecular Modeling and MD Simulations

The features of the active site of VldE^MT3^ are consistent with those of M15 metallopeptidases and their catalytic mechanism^67^: (i) one Zn^2+^ ion ligated by the side chains of two histidine residues (H301 and H375) and one aspartate (D308), and (ii) an arginine (R294), which is presumed to stabilize the negative charge on the transition-state species for the reaction. Nonetheless, neither the positioning of the nucleophilic water molecule nor its activation mechanism is immediately apparent from the crystallographic structures. The protonation states of H301 (on the N^δ^ atom) and H375 (on N^ε^) are unambiguous due to the coordination geometries of their respective free-base imidazole rings with the catalytic Zn^2+^ ion.

**Figure 3:**
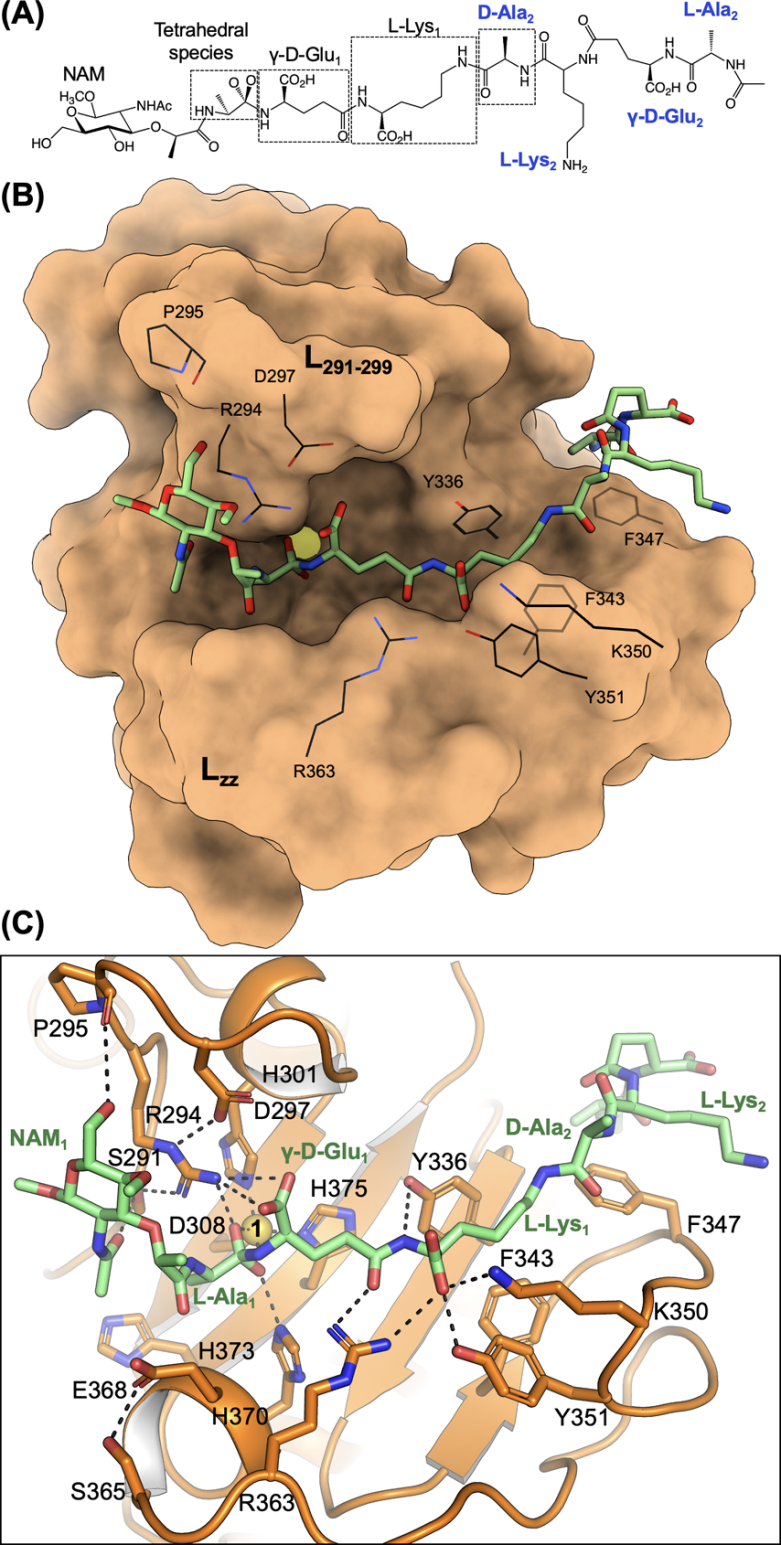
(A) Chemical scheme of the compound **8**-TS with the orientation shown in panel B. Residues originating from different strains are labeled and distinguished by varying colors, black and blue. (B) Modeled substrate recognition by VldE^MT3^. The protein is represented as an orange surface, and the substrate is shown as capped sticks colored by atom types (green for carbon). Residues involved in substrate recognition are superimposed on the surface and labeled. (C) Zoomed-in view of the modeled interactions between VldE^MT3^ and the TS. The zinc cation is represented as a yellow sphere.

The structural comparison with the related M15 and M23 family peptidases (Figure S17), as observed in the VanXYg: d-Ala-d-Ala phosphinate complex (PDB id. 4MUQ),^45^ allowed us to provide a transition-state model for VldE^MT3^ (Figure 3) that remained stable over the simulation time (300 ns) (Figure S19A). This model explains how cross-linked peptide stems can be accommodated into the VldE^MT3^ active site: (i) the tetrahedral species for the transition state is sequestered by the guanidinium of R294, the carboxylate of D308 and the imidazolium ring of H370 (Figure 3C), and (ii) the enzyme’s specificity would appear to be determined by a small pocket that accommodates the l-Ala side-chain methyl (comprised of the side chains of S291, M310, T367, and H373) and by electrostatic interactions involving the carboxylates of γ-d-Glu and l-Lys (Figure 3C). Thus, the free α-carboxyl of γ-d-Glu forms a salt bridge with the guanidinium of R294 (in case this position is occupied by γ-d-Gln *H*-bond interaction could also be feasible), whereas the free α-carboxylate of l-Lys establishes salt-bridge interactions with R363 and K350, and hydrogen-bonding interaction with the side chain of Y351. R363 also binds to the main-chain carbonyl of d-Glu (Figure 3C and Movies S2–S4).

The proposed sequestration of the γ-d-Glu-l-Lys peptide bond would allow the proper orientation of the asymmetric substrate by recognition of the unique l-Lys α-carboxylate in the acceptor strand of the cross-linked peptidoglycan. The orientation of the transition state observed in this model is consistent with VldE^MT3^ binding to the cross-linked peptide stem (Figure S19C), a finding that accounts for the observed substrate specificity.

## Discussion

The TCS VicRK has been documented to play an essential role in *S. pneumoniae* by coordinating various cellular processes crucial for the survival and pathogenesis of this bacterium.^68^ The significance of this regulon is underscored by the extensive characterization of its members and their roles in pneumococcal physiology.^7−11^ The expression of the VicRK-regulated gene *spr1875*, known to be induced upon cell-wall stress,^15^ was validated in this study (Figure S1). Furthermore, this study demonstrated that VldE expression is also induced under other cell stress conditions (Figure 1B). However, the precise function and role of the Spr1875 protein remained elusive heretofore. The present work shows that the induced protein localizes to septa in stressed cells and decreases the susceptibility of pneumococcal cells subjected to penicillin stress. Furthermore, we documented by mass spectrometric analyses of the turnover chemistry of suitable synthetic peptidoglycan-based substrates that the full-length enzyme and the MT3 catalytic domain exhibit an l,d-endopeptidase activity (hydrolysis of the cross-linked stem peptides at a specific bond). This activity has been reported to play a crucial role for cell viability in *Staphylococcus aureus*.^69^

**Figure 4:**
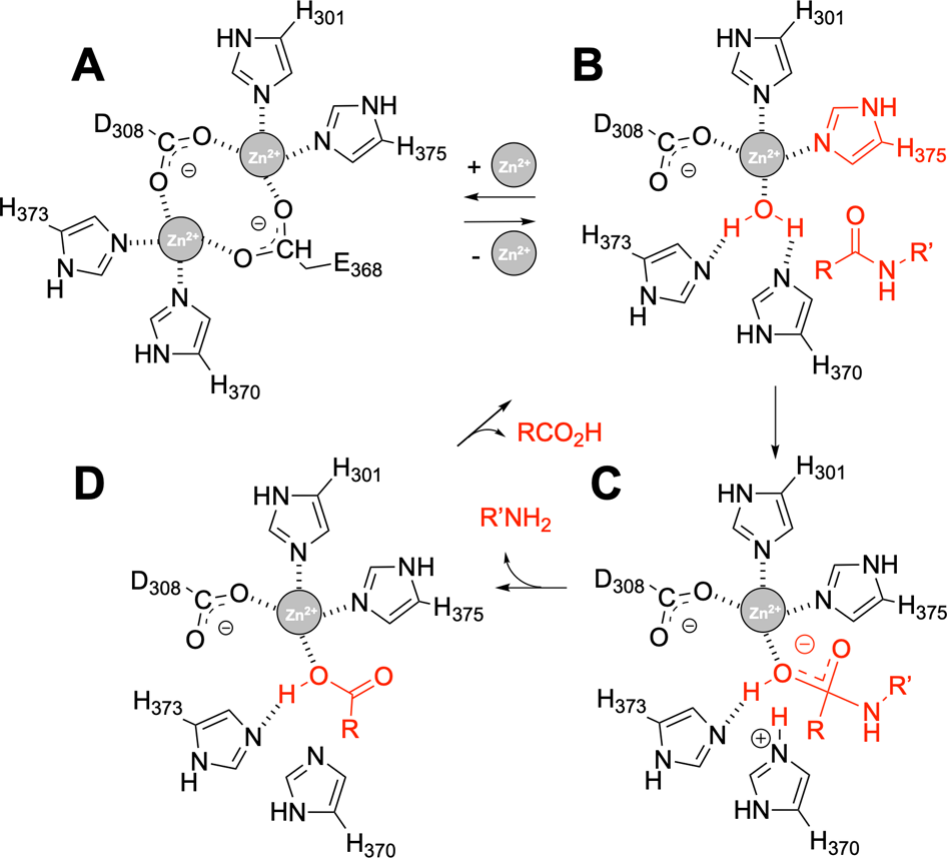
Proposed catalytic cycle of VldE^MT3^. (A) Inhibited state is depicted with zinc ions 1 and 2 in place. (B) Dissociation of zinc 2 reorganizes the active site to place H370 as the base that promotes the hydrolytic water molecule to form the transition-state species (C). Collapse of the transition state leads to the formation of the two products of the reaction (panels C and D) and a return to the ground state active form (B).

The folding space of VldE^MT3^ was thoroughly explored after crystallization under different zinc-availability conditions and MD simulations. The X-ray structure of the protein showed an unprecedented in-line, four-zinc-ion binding site, which, to the best of our knowledge, has not been described previously for any protein. The function of the four-zinc ion motif is not fully understood at present. It may have a mechanistic implication as a reservoir of zinc ions, since we added no exogenous zinc ions, nor to the crystallographic buffers (while the presence of zinc ions cannot be discarded in a growth medium). Zinc is an essential nutrient metal required for the catalytic activity or structural stability of thousands of proteins. During infections, hosts use both zinc starvation and toxicity as strategies to limit microbial survival.^70,71^ Therefore, metal homeostasis systems are required for pathogen survival within the host. In support of this hypothesis, there are multidomain proteins (mainly from environmental bacteria *Actinobacteria*) that include VldE^MT3^ homologues combined with lysozyme-like, SH3 and/or peptidase domains, whose function is associated in some cases with zinc ions (Figure S20).^72−75^ The attachment of the four zinc ions within the active site prevents substrate binding, hence abrogating the activity. Nonetheless, the endopeptidase activity of VldE is likely attributable to a single catalytic zinc (Zn-1), based on precedent from related enzymes.^76^ A latent state has been reported for other metalloproteases, such as the zinc-ligand switch described for the *S. aureus* LytM^53^ in which N117 blocks the zinc ion and replaces the catalytic water. The multizinc-binding structure reported in the present work may represent a latent state of VldE, with the E368 residue blocking the catalytic zinc ion. Reversal of this coordination would be the onset of activation from the latent form (Figure 4). In line with this reasoning, our zymogram experiments confirmed that E368 was not essential for catalysis, as the muralytic activity was retained in the E368A variant. Regulatory mechanisms for other zinc-dependent enzymes have already been reported: (i) binding of a second zinc ion causes the spatial rearrangement of catalytic histidines, decreasing *Staphylococcus simulans* lysostaphin catalytic activity,^77^ and (ii) a pH-dependent inhibitory effect on LytU endopeptidase from *S. aureus* has been observed.^78^ Remarkably, a structural reorganization in the β6-β7 loop of LytU (similar to the L_zz_ region of VldE^MT3^) was observed among structures containing either one or two bound Zn^2+^ ions, closing the active-site cleft in the two-zinc form.^78^

The VldE^MT3^|_act_ structure, with a single zinc ion, is likely to represent a catalytically competent state of the protein that can nicely accommodate the cross-linked peptidoglycan (Figures 3 and S19C) while the specific events triggering activation remain unknown. The water molecule identified in this structure (Figure 2G) is proposed to mediate -upon activation by H370- hydrolysis by a nucleophilic attack on the peptide bond (Figure 4), as also reported for the DCC peptidase from *Streptomyces albus* in^56^ and LytM of *S. aureus*.^72^ We note that residues H370 and H373 (Figure S6) are conserved in other orthologs (Figure S17A–C). In agreement with their assigned role, mutation of H345 residue (equivalent to H370 in VldE) abrogates activity in the ortholog ShyA from *Vibrio cholerae*.^65^

The coordinated Zn^2+^ ion and R294 contribute to the polarization of the carbonyl group in the scissile amide bond. The conserved residue R294, guided by its hydrogen bond with S291, is likely to stabilize the negative charge on the tetrahedral species.^56,66^ The essentiality of this role was confirmed in the d,d-peptidase VanXYg from *Enterococcus faecalis*, as the mutation of the corresponding residues, S71 and R74, abrogated its activity.^45^ R294, whose guanidinium group is fixed by the carboxylate of D297, appears to play an additional role in the recognition of the α-carboxyl group of d-Glu, hence contributing to substrate specificity. The free α-carboxylate of l-Lys, defining the orientation of the acceptor strand in the cross-linked peptidoglycan, likely plays a role in selectivity by its interactions with K350, Y351, and R363 (Figure 3C).

## Conclusions

In summary, we have shown that VldE can act as a highly regulated protein with two structural states. The structure of the protein presents an unprecedented zinc-ion-binding capacity in its inactive form and an l,d-endopeptidase activity in its active state. Further work will be required to unveil how VldE activation is produced and how these two states contribute to the regulation of cell-wall remodeling during stress of the human pathogen *S. pneumoniae*.

## Supporting Information Available

Antibiotics induces expression of Spr1875-sfGFP; functionality of Spr1875-sfGFP; localization of Spr1875-sfGFP; growth and cell size parameters of WT and Dspr1875 (vldE) cells; growth of S. pneumoniae penicillin-sensitive strain R6 treated with increasing concentrations of vancomycin or ciprofloxacin; muralytic activity of CHiC-Spr1875MT3 (VldEMT3) wild-type and altered versions; chemical structures of the synthetic PG fragments used for the assessment of substrate specificity; intact protein analysis of three different constructs of Spr1875 (VldE) by LC/MS; reactions of three constructs of Spr1875 (VldE) and compounds; distribution of sedimenting species for the VldE^MT3^ domain by analytical ultracentrifugation; topology diagram of the VldE^MT3^ domain with a four-zinc cluster; anomalous-difference-Fourier map indicating peaks for zinc cations inside VldE^MT3^ groove; structural homologues to the VldEMT3 domain include d,d-carboxypeptidases and endolysins from bacteriophages; thermal-conformational transitions studied by intrinsic fluorescence; superimposition of the catalytically competent VldEMT3 active site with the homologues; AlphaFold2 predicted structure of VldEMT3 domain; peptidoglycan recognition by VldEMT3; and distribution and domain composition of VldE^MT3^ homologs (PDF)
Representative molecular dynamics trajectory of the apo form of VldE (MP4)

## Abbreviations

CID: collision-induced dissociation
GFP: green fluorescent protein
LC/MS: liquid chromatography–mass spectrometry
LAS: lysostaphin-type enzymes, d-Ala-d-Ala metalloproteinases and the Zn-dependent peptidase of the sonic hedgehog protein
MD: molecular dynamics
L_zz_: zinc-zipper loop
MS/MS: tandem mass spectrometry
MT3: motif 3 present in the TM4 tape measure protein
PenG: penicillin G
PG: peptidoglycan
TCS: two-component system
TS: transition state.
