# Supplementary material for: Characterization of VldE (Spr1875), a Pneumococcal Two-State l,d-Endopeptidase with a Four-Zinc Cluster in the Active Site
Source: ACS Catal. 2024 Dec 11;14(24):18786–98. doi: 10.1021/acscatal.4c05090 (PMC11667670; doi:10.1021/acscatal.4c05090)
Supplement: Supplementary file 1 — cs4c05090_si_001.pdf [file cs4c05090_si_001.pdf]

Supporting Information

Characterization of VldE (Spr1875), a pneumococcal two-state L,D-endopeptidase with a four-zinc cluster in the active site

Vega Miguel-Ruano[1,#], Iván Acebrón[1,#], Mijoon Lee[2], Antonio J. Martín-Galiano[3], Celine Freton[4], Uxía P. de José[1], Balajee Ramachandran[2], Federico Gago[5], Morten Kjos[6], Dusan Hesek[2], Christophe Grangeasse[4], Leiv Sigve Håvarstein[6], Daniel Straume[6], Shahriar Mobashery[2*] and Juan A. Hermoso[1*]

[1]Department of Crystallography and Structural Biology, Instituto de Química-Física "Blas Cabrera", Consejo Superior de Investigaciones Científicas, Madrid 28006, Spain. [2]Department of Chemistry and Biochemistry, University of Notre Dame, Notre Dame, IN 46556, USA. [3]Core Scientific and Technical Units, Carlos III Health Institute, Majadahonda 28222, Madrid, Spain. [4]Molecular Microbiology and Structural Biochemistry, CNRS UMR, Université de Lyon, Lyon 69367, France. [5]Department of Biomedical Sciences & IQM-CSIC Associate Unit, School of Medicine and Health Sciences, University of Alcalá, Alcalá de Henares 28805, Spain. [6]Department of Chemistry, Biotechnology and Food Science, Norwegian University of Life Sciences, Ås 1430, Norway.

[#]Equally contributing authors

*Corresponding Authors: : mobashery@nd.edu and xjuan@iqfr.csic.es

Table of Contents

Supplementary Figures S2

Supplementary Movies S25

Supplementary Tables S26

References S32

Supplementary Figures

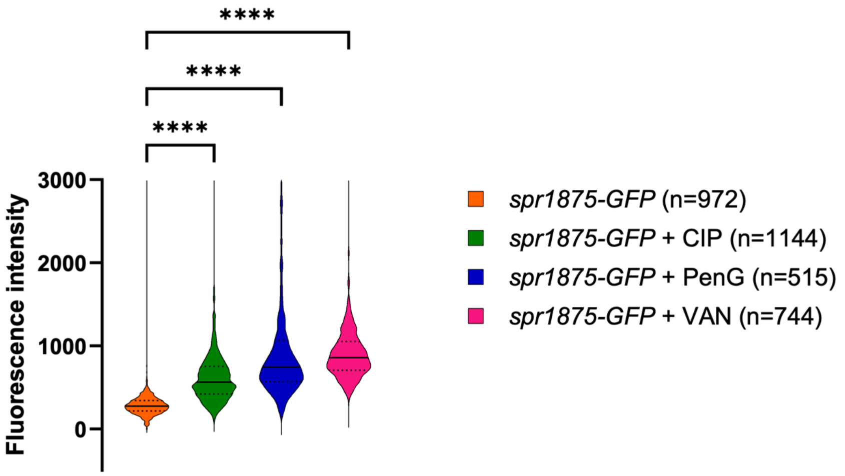

Figure S1. Antibiotics induces expression of Spr1875-sfGFP. Total fluorescence signal of Spr1875-sfGFP cells after treatment with 0.025 µg/mL of Penicillin G (PenG) or 0,25µg/mL of Ciprofloxacin (CIP) or 0,25µg/mL of Vancomycin (VAN). The means is indicated with a black line. The 25th and 75th percentile are indicated with a dash line. The number of cells (n) analyzed is indicated on the right. Representative of experiments made in triplicate. Statistical comparison was done using Kruskal – Wallis test and Dunn's Multiple comparaison test . ****p < 0.0001.

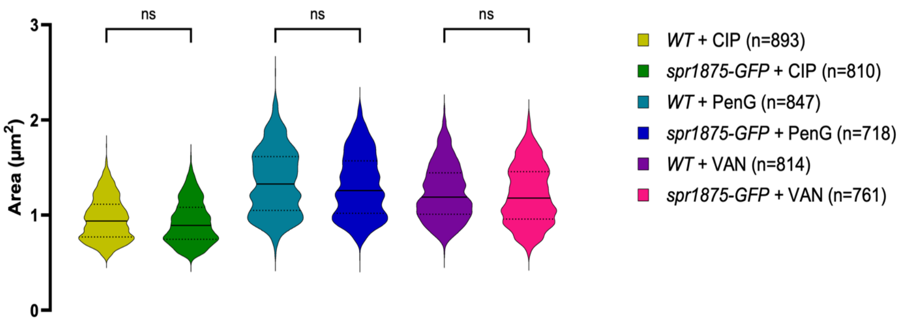

Figure S2. Functionality of Spr1875-sfGFP. Violin plot showing the distribution of the cell area (µm²) measured for WT (R6) and *spr1875-sfGFP* cells after treatment with 0,025 µg/mL of Penicillin G (PenG) or 0,25µg/mL of Ciprofloxacin (CIP) or 0,25µg/mL of Vancomycin (VAN). The means is indicated with a black line. The 25th and 75th percentile are indicated with a dash line. The number of cells (n) analyzed is indicated on the right. Representative of experiments made in triplicate. Statistical comparison was done using Kruskal – Wallis test and Dunn's Multiple comparaison test . ns : non significant.

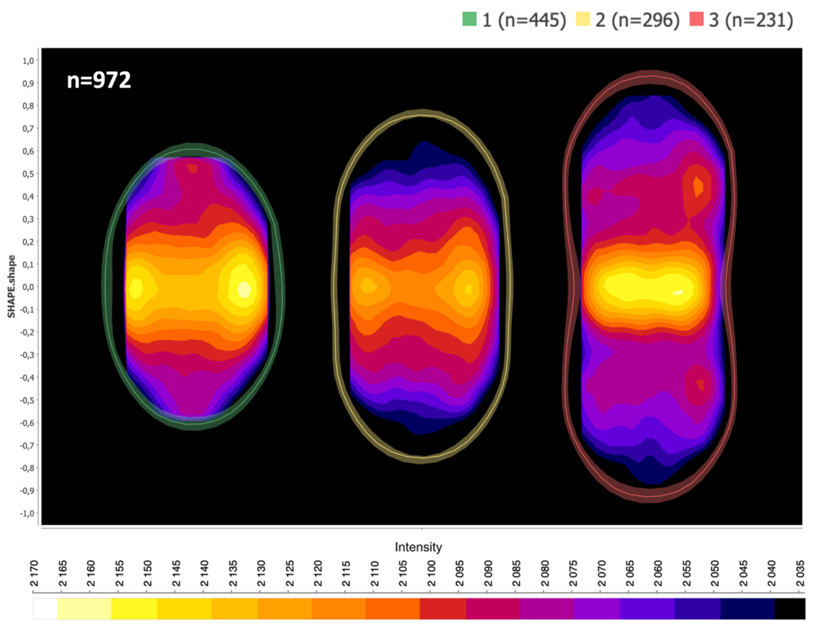
1 (n=445) 2 (n=296) 3 (n=231)
n=972
SHAPE.shape
1,0 0,9 0,8 0,7 0,6 0,5 0,4 0,3 0,2 0,1 0,0 -0,1 -0,2 -0,3 -0,4 -0,5 -0,6 -0,7 -0,8 -0,9 -1,0
Intensity
2 170 2 165 2 160 2 155 2 150 2 145 2 140 2 135 2 130 2 125 2 120 2 115 2 110 2 105 2 100 2 095 2 090 2 085 2 080 2 075 2 070 2 065 2 060 2 055 2 050 2 045 2 040 2 035

+ CIP

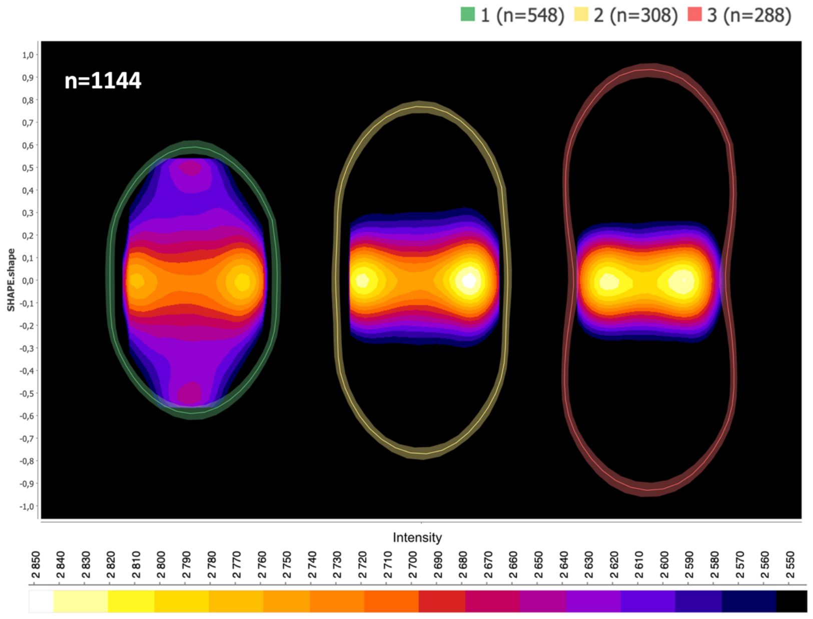
1 (n=548) 2 (n=308) 3 (n=288)
n=1144
SHAPE.shape
1,0 0,9 0,8 0,7 0,6 0,5 0,4 0,3 0,2 0,1 0,0 -0,1 -0,2 -0,3 -0,4 -0,5 -0,6 -0,7 -0,8 -0,9 -1,0
Intensity
2 850 2 840 2 830 2 820 2 810 2 800 2 790 2 780 2 770 2 760 2 750 2 740 2 730 2 720 2 710 2 700 2 690 2 680 2 670 2 660 2 650 2 640 2 630 2 620 2 610 2 600 2 590 2 580 2 570 2 560 2 550

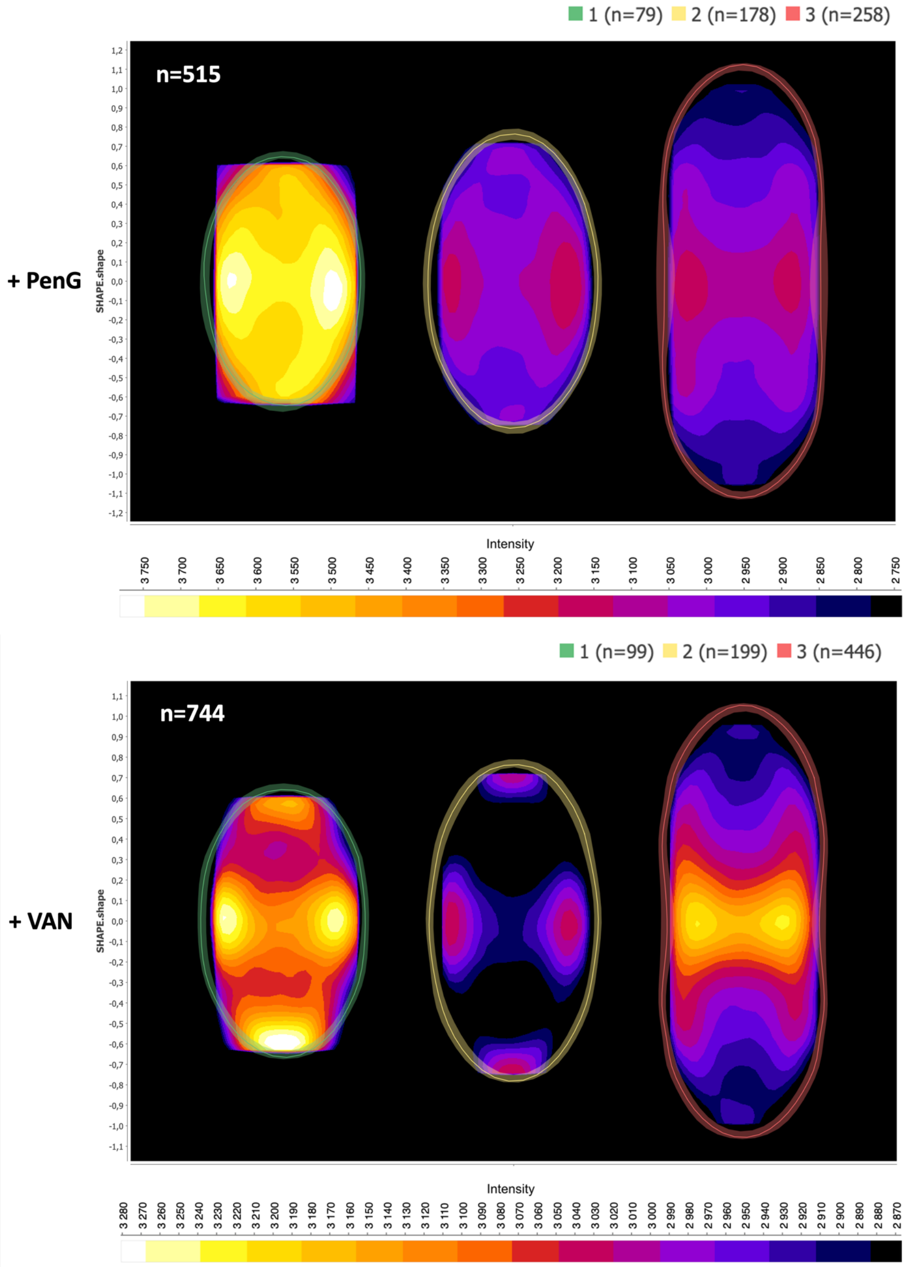
+ PenG
1 (n=79) 2 (n=178) 3 (n=258)
n=515
SHAPE.shape
1,2 … -1,2
Intensity
3 750 3 700 3 650 3 600 3 550 3 500 3 450 3 400 3 350 3 300 3 250 3 200 3 150 3 100 3 050 3 000 2 950 2 900 2 850 2 800 2 750
+ VAN
1 (n=99) 2 (n=199) 3 (n=446)
n=744
SHAPE.shape
1,1 … -1,1
Intensity
3 280 3 270 3 260 3 250 3 240 3 230 3 220 3 210 3 200 3 190 3 180 3 170 3 160 3 150 3 140 3 130 3 120 3 110 3 100 3 090 3 080 3 070 3 060 3 050 3 040 3 030 3 020 3 010 3 000 2 990 2 980 2 970 2 960 2 950 2 940 2 930 2 920 2 910 2 900 2 890 2 880 2 870

Figure S3. Localization Spr1875-sfGFP. Heat maps showing the localization of Spr1875-sfGFP in absence or presence of Penicillin G (PenG), Ciprofloxacin (CIP) or Vancomycin (VAN). n indicates the number of cells analyzed. Representative of experiments made in triplicate.

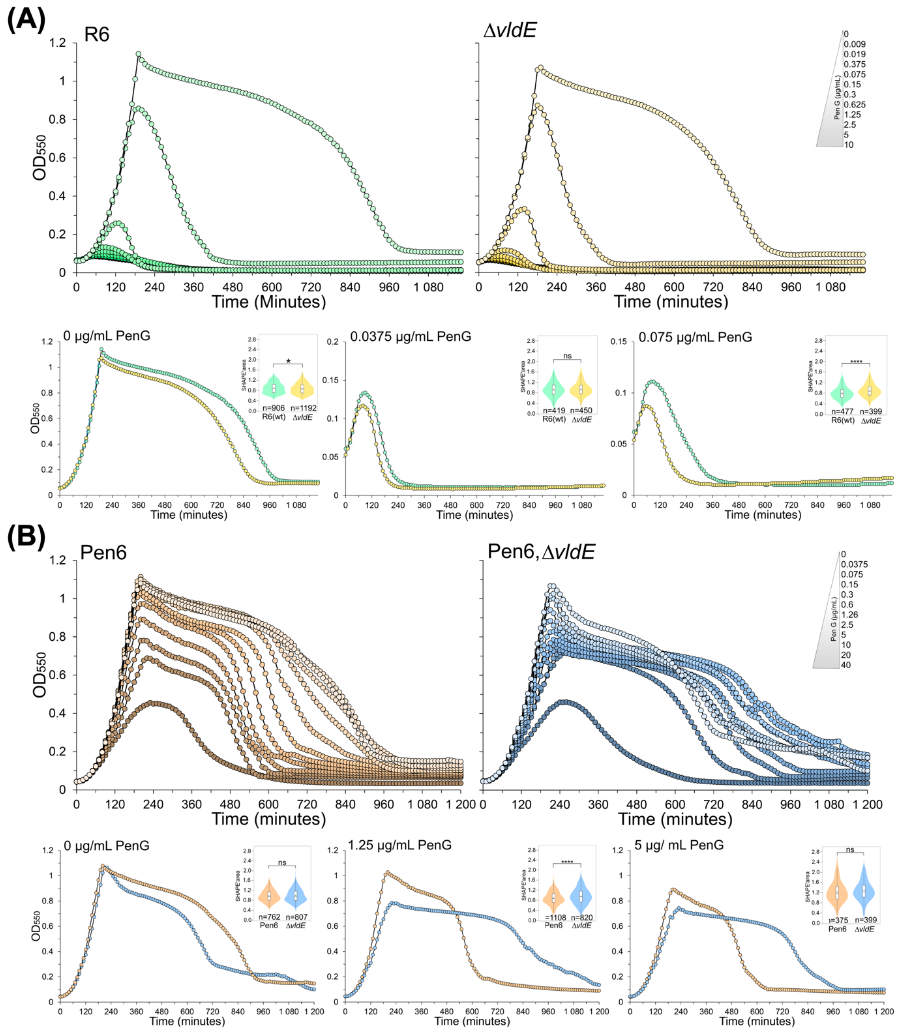

Figure S4. **Growth and cell size parameters of WT and Δ*spr1875* (*vldE*) cells.** Analysis were performed in R6 and Pen6 strains, either in absence or presence of different concentrations of penicillin G. The growth patterns of wild type and Δ*spr1875* (*vldE*) mutants are shown for the R6 (A) and Pen6 (B) strains exposed to different penicillin G concentrations. In the penicillin resistant Pen6 strain, deletion of *spr1875* (*vldE*) resulted in reduced growth and a significantly delayed

onset of autolysis (2.5 - 4 hours) upon penicillin G exposure (0.075 – 5 µg/mL) compared to the parental strain. The bottom panels (A and B) present selected growth curves for the R6 and Pen6 strains repectively, displaying difference in growth between wild type and the Δ *spr1875* (*vldE)* mutants. Wild type R6 and Pen6 are presented in green and red, respectively, and their Δ *spr1875* (*vldE*) derivates in yellow and blue, respectively. For microscopy, cells at OD_{550} = 0.1 were added the respective penicillin G concentrations and grown for 2 hours before microscopic imaging. Cell size distribution is presented as violin plots. For R6, the average cell size of wild type was 0.89 ± 0.21 μm^2 and 0.87 ± 0.21 μm^2 for the Δ *spr1875* (*vldE*) mutant (low statistical significance, P= 0.015). At 0.075 µg/mL penicillin G wild-type cells had an average size of 0.80 ± 0.23 μm^2 compared to 0.90 ± 0.24 μm^2 for the Δ *spr1875* (*vldE*) cells. For the Pen6 strain a significant difference in average cell size was observed at 1.25 µg/mL penicillin G. Wild type having an average size of 0.88 ± 0.26 μm^2 compared to 0.96 ± 0.32 μm^2 for the Spr1875 (VldE)-deficient cells. P values were obtained relative to wild type using one-way analysis of variance, *P<0.05, ****P < 0.0001.

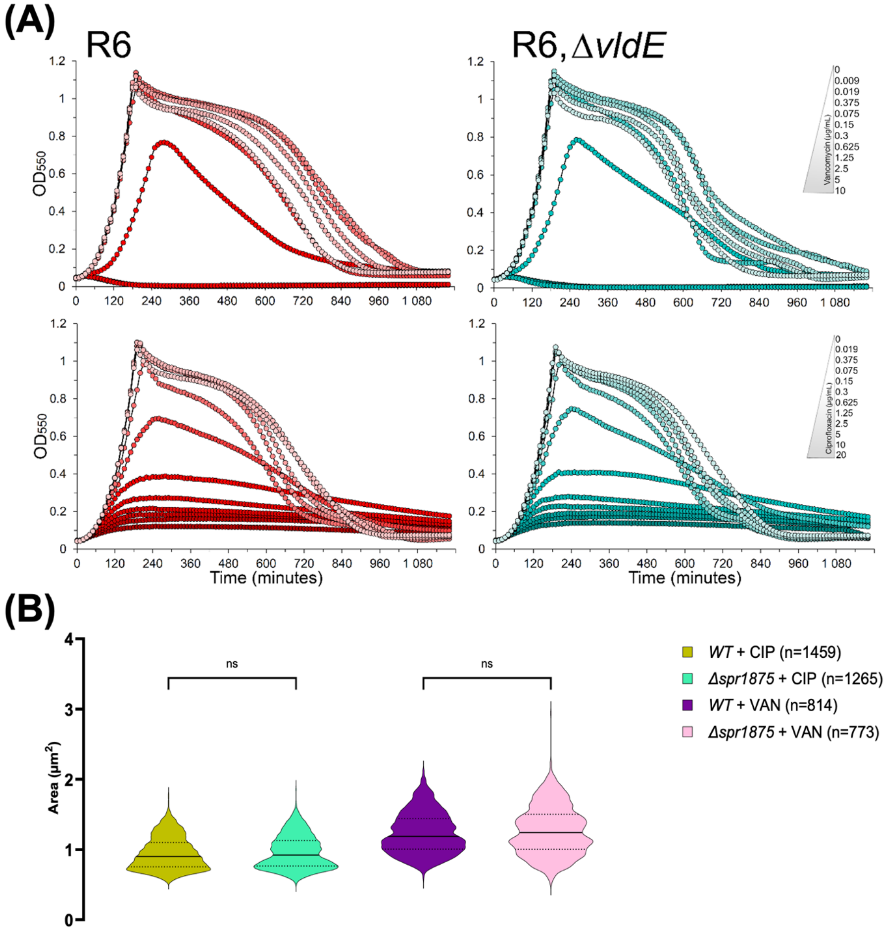

Figure S5. (A) Growth of *S. pneumoniae* penicillin sensitive strain R6 treated with increasing concentrations of vancomycin or ciprofloxacin. Growth of VldE proficient cells is shown in red and VldE deficient mutants in cyan. **(B)** Impact of the deletion of *spr1875*. Violin plot showing the distribution of the cell area (µm²) measured for WT (R6) and Δ*spr1875* cells after treatment with 0,25µg/mL of Ciprofloxacin (CIP) or 0,25µg/mL of Vancomycin (VAN). The means is indicated with a black line. The 25th and 75th percentile are indicated with a dash line. The number of cells (n) analyzed is indicated on the right. Representative of experiments made in triplicate. Statistical comparison was done using Kruskal – Wallis test and Dunn's Multiple comparaison test . ns : non significant.

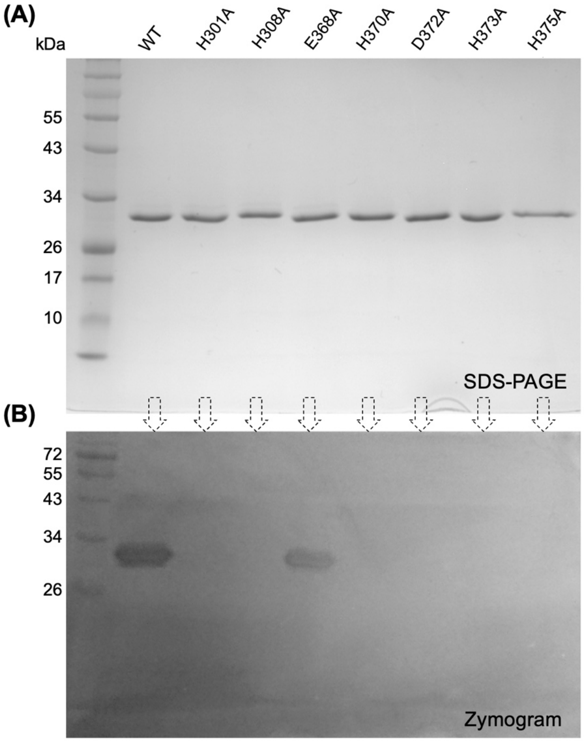

Figure S6. Muralytic activity of CHiC-Spr1875^{MT3} (VldE^{MT3}) wild-type and altered versions. Panel **(A)** includes an SDS-PAGE gel showing the purity of Spr1875^{MT3} (VldE^{MT3}) wild-type (WT) and of variants in conserved residues. Panel **(B)** presents a zymogram analysis for the same protein variants. The first lane contains a molecular-weight marker, with molecular-mass standards in kDa indicated. The WT protein showed muralytic activity. Residues H301, D308 and H375 are predicted to participate in metal coordination,[1] and muralytic activity was lost when these amino acids were changed to alanine individually. Amino acids H370, D372 and H373 are highly conserved among proteins containing the MT3 motif,[2] and their modifications to alanine also rendered the enzyme inactive. Conversely, alteration of the adjactent residue E368 did not affect the activity.

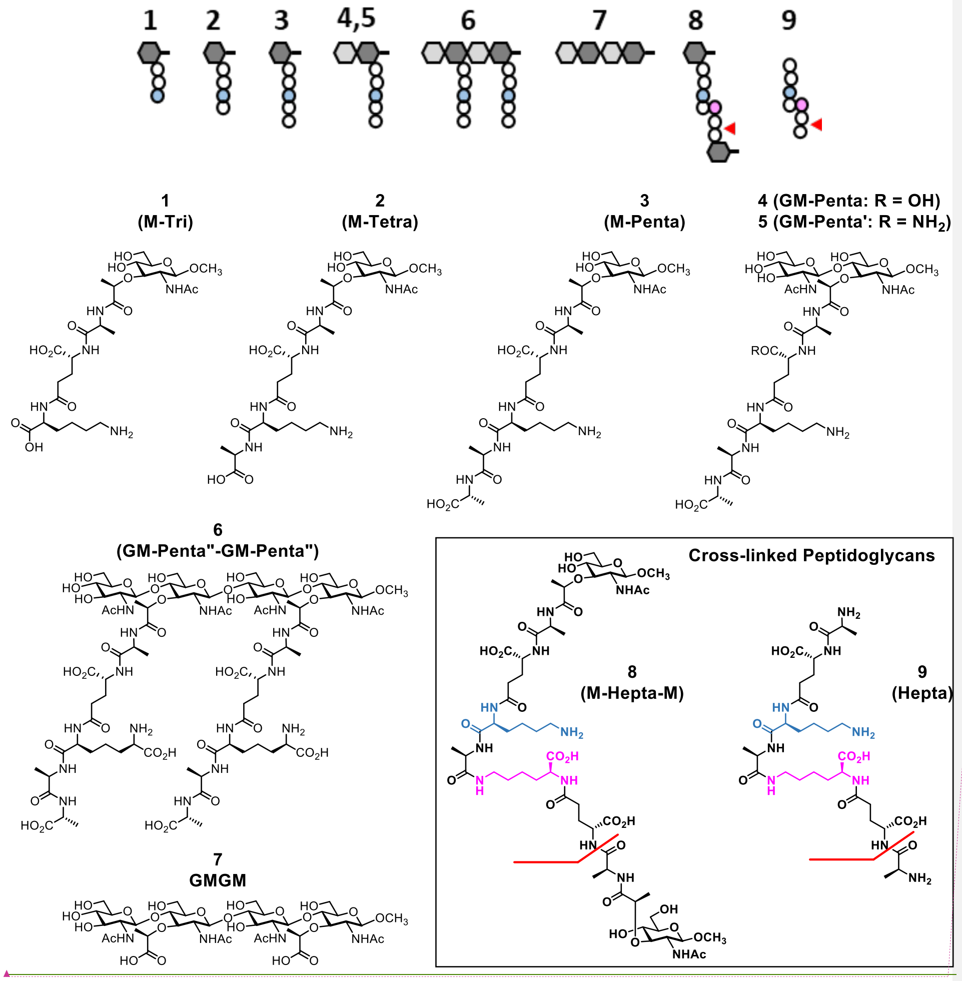

Código de campo cambiado

Figure S7. Chemical structures of the synthetic PG fragments used for the assessment of substrate specificity of Spr1875 (VldE), CHiC-Spr1875^{MT3} (VldE^{MT3}) and Spr1875^{MT3} (VldE^{MT3}) (1-9). M stands for 1-*O*-β-methyl *N*-acetyl-muramic acid and G, for *N*-acetyl-glucosamine. The full sequence of the pentapeptide ("Penta**", 3** and **4**), is L-Ala-γ-D-Glu-L-Lys-D-Ala-D-Ala. D-Gln in Penta' (**5**) substitutes for D-Glu, a common modification found in *S. pneumoniae* cell wall; L-Lys in Penta'' (**6**) was replaced with *m*-DAP. 4-3 Cross-linked PG

fragments (D-Ala-L-Lys), **8** and **9**, are represented inside a black frame. The red lines and triangles indicate the inferred Spr1875 (VldE) hydrolytic cleavage site.

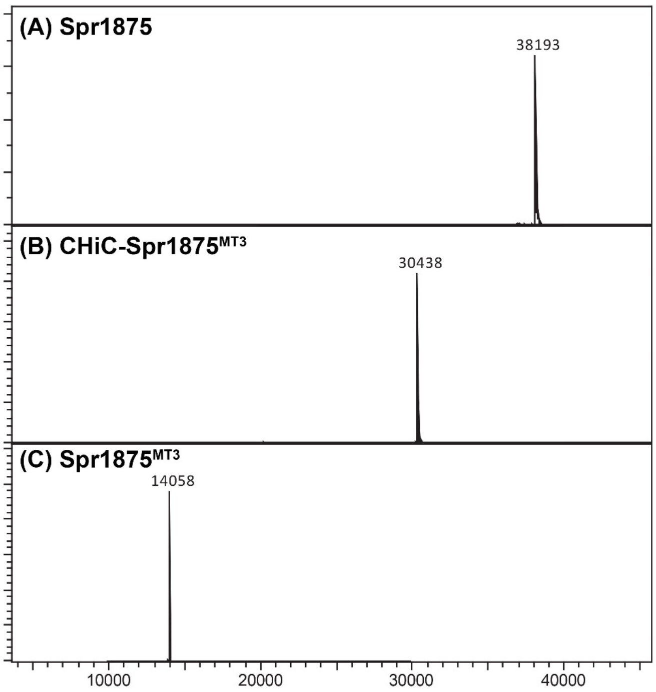

Figure S8. Intact protein analysis of three different constructs of Spr1875 (VldE) by LC/MS. The deconvoluted mass spectra of **(A)** Spr1875, **(B)** CHiC-Spr1875^{MT3} and **(C)** Spr1875^{MT3}.

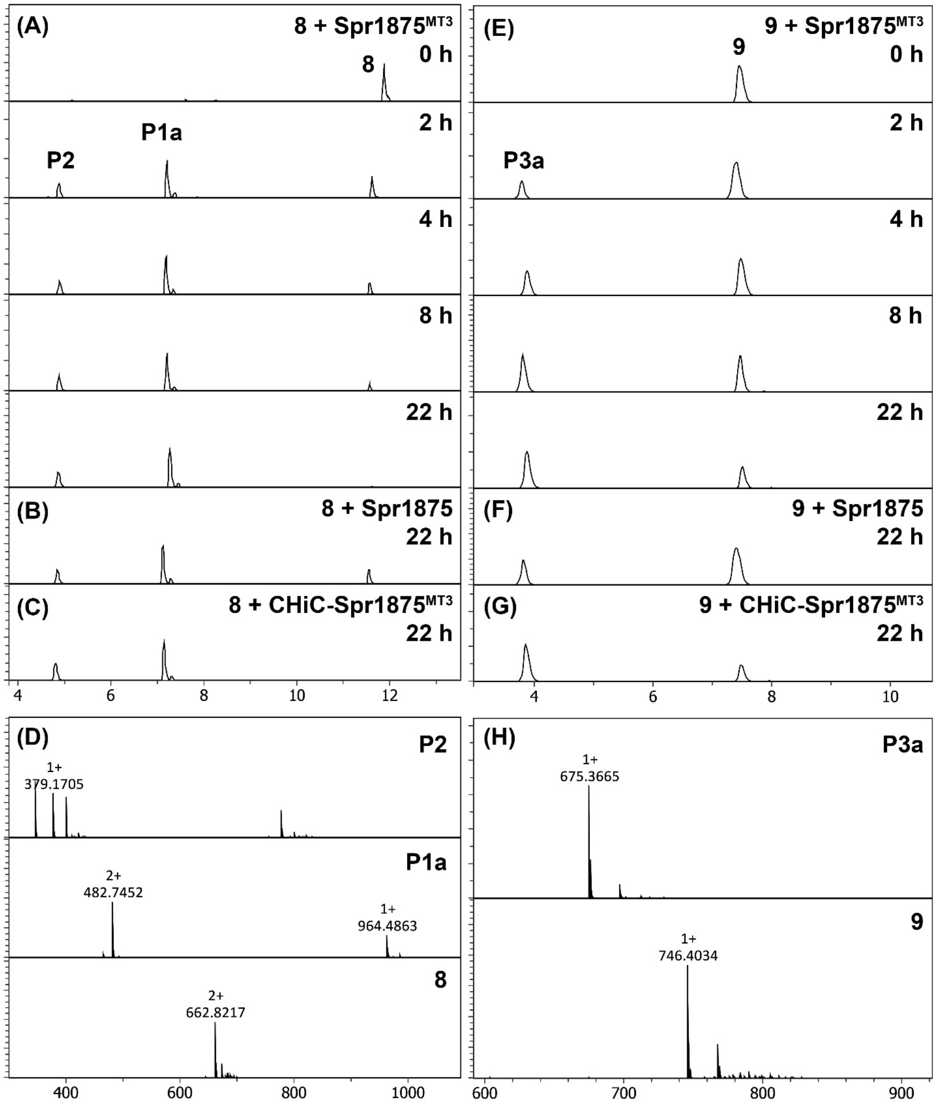

Figure S9. Reactions of three constructs of Spr1875 (VldE) and compounds **(A-D) 8** and **(E-H) 9**. **(A-C, E-G)** the LC/MS traces and **(D, H)** the mass spectra of substrates and reaction products. The *m*/*z* values with +1 shown in mass spectra are $[M+H]^+$ and those with +2 are $[M+2H]^{2+}$.

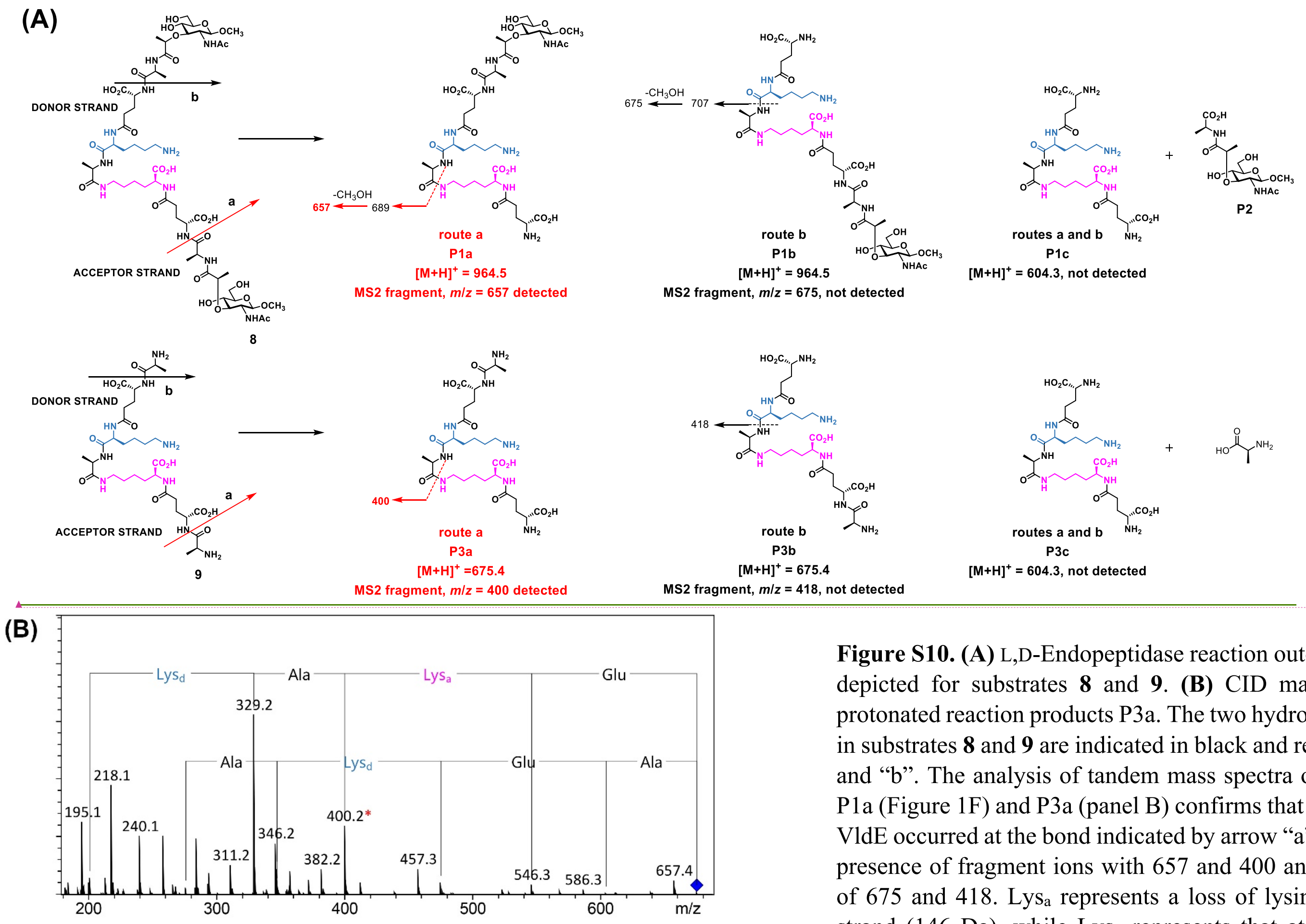

Figure S10. (A) L,D-Endopeptidase reaction outcome of VldE depicted for substrates **8** and **9**. **(B)** CID mass spectra of protonated reaction products P3a. The two hydrolysable bonds in substrates **8** and **9** are indicated in black and red arrows, "a" and "b". The analysis of tandem mass spectra of the product P1a (Figure 1F) and P3a (panel B) confirms that hydrolysis by VldE occurred at the bond indicated by arrow "a" based on the presence of fragment ions with 657 and 400 and the absence of 675 and 418. Lys_a represents a loss of lysine at acceptor strand (146 Da), while Lys_d represents that at donor strand (128 Da).

Código de campo cambiado

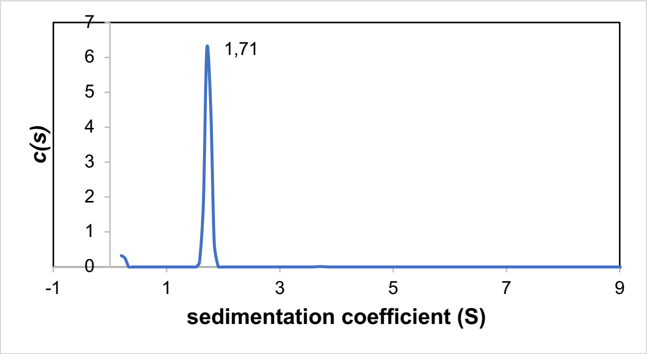

Figure S11. Distribution of sedimenting species for the VldE^{MT3} domain by analytical ultracentrifugation. The calculated sedimentation coefficient distribution, c(s), was plotted as a function of the sedimentation coefficient (S). The distribution reveals the presence of one major species, with an *S* value of 1.71 corresponding to a molecular weight close to the apparent weight for the monomeric domain.

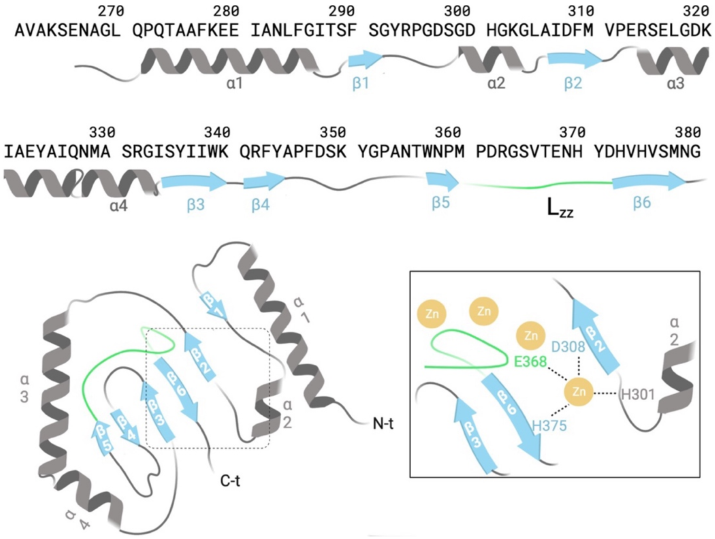

Figure S12. Topology diagram of the VldE^{MT3} domain with a four-zinc cluster. The top section displays the protein sequence and secondary structure, with α–helices represented in gray and β-strands in light blue. The L_{zz} loop is highlighted in green. The bottom section presents a topology diagram of the VldE^{MT3} domain, showing a LAS arrangement. An inset provides a detailed view of the four-zinc-binding site. The figure was prepared using BioRender.

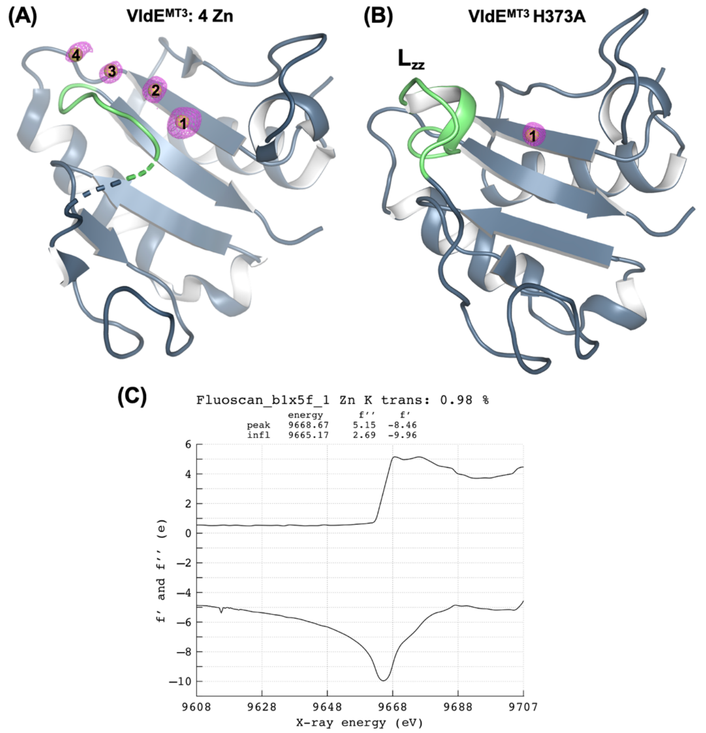

Figure S13. (A-B) Anomalous-difference-Fourier map indicating peaks for zinc cations inside VldE^{MT3} groove. VldE^{MT3}crystals were measured at the K-edge of zinc, 1.282Å, and anomalous maps were generated as a pink mesh, contoured at 4.0 σ and superposed on the structure. The protein structure is represented in blue cartoon with the L_{zz} region highlighted in green. Zinc cations are represented as yellow spheres and numbered according to their position in the cavity. **(A)** Four anomalous densities were observed within the VldE^{MT3}:4 Zn structure. **(B)** The VldE^{MT3} H373A structure showed a unique peak for zinc-1. **(C)** X-ray fluorescence scan measuring intensity as a function of X-ray energy (eV) for VldE^{MT3}:4 Zn crystals showed absorption at the zinc energy edge and confirmed the presence of zinc.

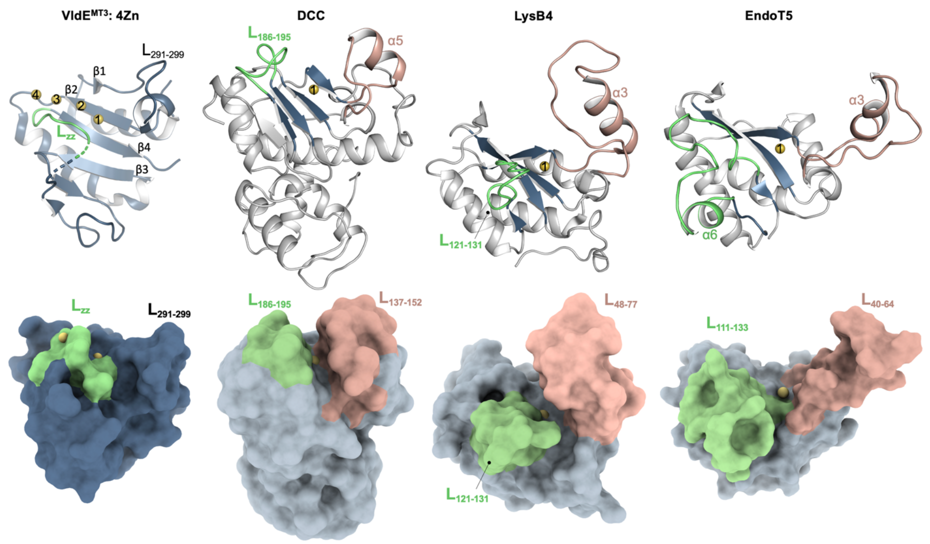

Figure S14. Structural homologues to the VldEMT3 domain include d,d-carboxypeptidases and endolysins from bacteriophages. The closest homologues are represented both as ribbon structures and solvent-accessable surface representation using ChimeraX, including the metallopeptidase DCC from *S.albus* (PDB:1LBU)[3], LysB4 endolysin from *Bacillus cereus* bacteriophage B4 (PDB:6AKV)[4] and EndoT5 L-Ala D-Glu peptidase from *Escherichia* phage T5 (PDB:2MXZ)[5]. The LAS conserved arrangement is highlighted in blue in all VldE homologues. Zinc cations are represented as yellow spheres and numbered according to their position. The green color highlights the L_{zz} in VldE, as well as its corresponding regions in the homologous proteins. Additionally, a pink representation indicates the presence of an insertion between the first two beta-strands of the LAS folding, present in all $VldE^{MT3}$ homologues.

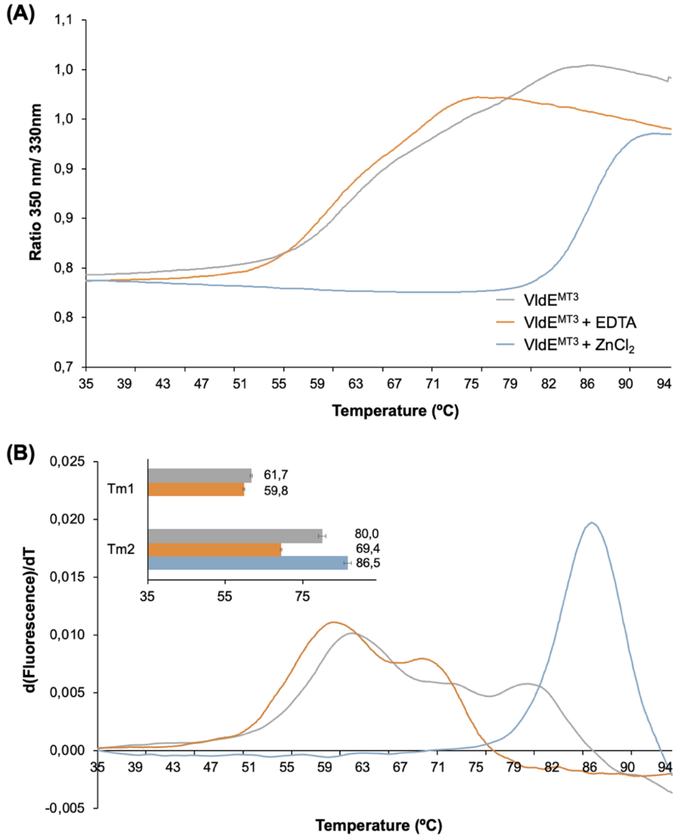

Figure S15. Thermal-conformational transitions studied by intrinsic fluorescence. (A) Melting-curve analysis (350nm/330nm fluorescence ratio) of VldE^{MT3} under different conditions is shown: VldE^{MT3} with no additives as a reference (gray), in the presence of 20 µM EDTA (orange), and in the presence of 20 µM of $ZnCl_2$ (blue). Thermal unfolding analysis revealed two inflection points for VldE^{MT3}, Tm1 and Tm2, suggesting that the protein suffers two unfolding transitions. EDTA-mediated removal of Zn^{+2} resulted in protein destabilization, causing a significant reduction in both Tm values, with Tm2 decreasing by 17.1 ºC. Conversely, supplementation with $ZnCl_2$ exerted a stronger stabilizing effect, prompting a shift into a single Tm of 86.5 ºC. **(B)** Corresponding thermal shifts (first derivative profiles) for the same conditions (same color scheme) are shown. The melting temperatures are represented in the inset, showing the average value and the error bars for the standard deviation, for n = 3.

(A)

4 Zn

Zn	Interactions: Protein atoms	WAT	ACT
1	5	-	-
2	3	2	-
3	3	1	-
4	3	-	2

1: square pyramidal (C5)
2: trigonal bipyramidal (C5)
3: tetrahedral (C4)

3 Zn

Zn	Interactions: Protein atoms	WAT
1	4	-
2	4	1
3	3	3

1: tetrahedral (C4)
2: square pyramidal (C5)
3: octahedral (C6)

2 Zn

Zn	Interactions: Protein atoms	WAT
1	5	1
2	4	1

1: octahedral (C6)
2: trigonal bipyramidal (C5)

1 Zn

Zn	Interactions: Protein atoms	WAT
1	4 + 1 (3.5Å)	Not seen

1: trigonal bipyramidal (C5)

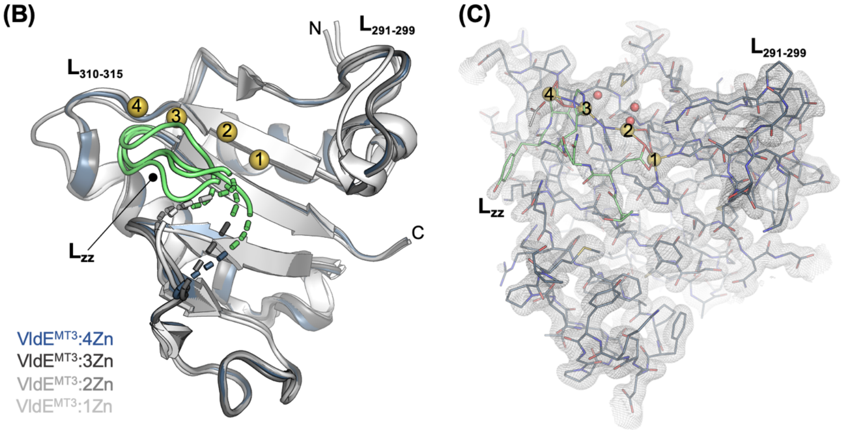

Figure S16. The tables in **(A)** include information on coordination geometry and zinc-ion-binding ligands for each VldE^{MT3} zinc-binding structure. **(B)** A three-dimensional superimposition of VldE^{MT3} zinc-binding structures is shown, with the VldE^{MT3}:4Zn structure displayed in dark-blue cartoon, and the other structures in varying shades of gray. The L_{zz} region is highlighted in green across all structures. Zinc cations are represented as yellow spheres, and regions with differences among the structures are labeled. **(C)** The $2F_o$-F_c electron-density map, contoured at 1σ, is superimposed onto the VldE^{MT3}:4Zn structure, which is shown as sticks. Zinc cations are represented as yellow spheres and water molecules coordinating the zinc atoms are represented as red spheres.

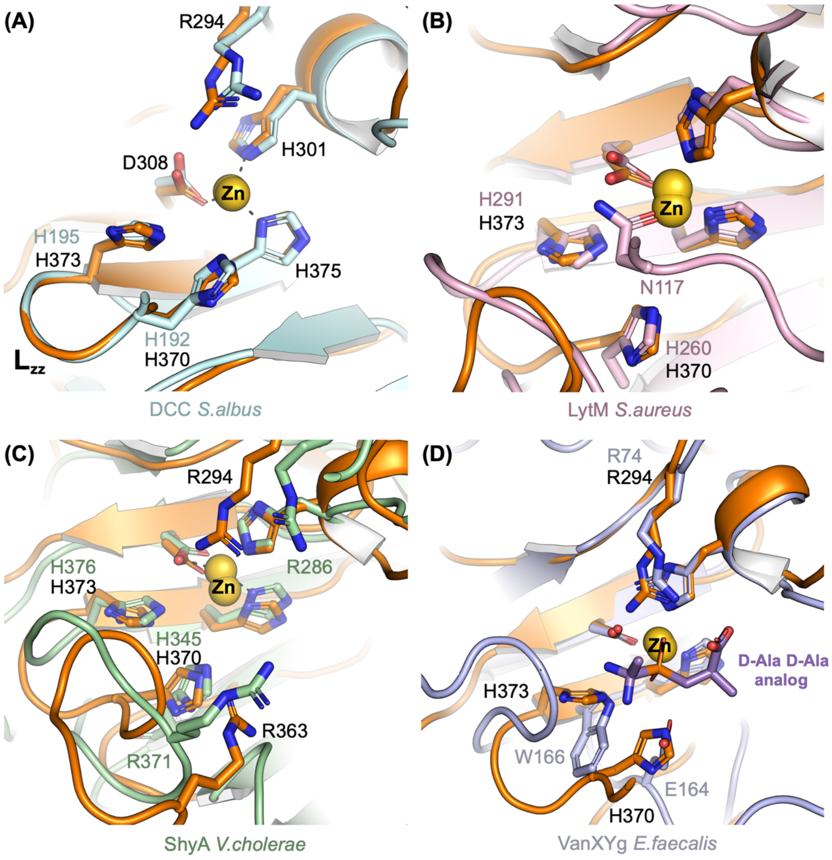

Figure S17 Superimposition of the catalytically competent VldEMT3 active site (displayed in orange ribbon) with the homologues. (A) DCC from *S. albus* (PDB: 1LBU; pale blue)[3], **(B)** LytM from *S. aureus* (PDB: 1QWY; light pink)[6], **(C)** ShyA from *V. cholerae* (PDB: 6U2A; pale green[7]), and **(D)** VanXYg from *E. faecalis* in complex with a D-Ala-D-Ala phosphinate substrate mimetic (PDB: 4MUQ; purple)[8]. Zinc cations are represented as yellow spheres and key residues within the active site are represented as sticks. The structural superimposition shows that N^{ε} of H373 in VldE is positionally and functionally equivalent to the N^{ε} nitrogens of H195 in DCC (PBB: 1LBU), H291 from LytM (PDB: 1QWY), H376 from ShyA (PDB: 6U2A) and to the non-basic 'pyrrole-like' indole nitrogens of W166 in VanXYg (PDB: 4MUQ), and W183 in VanX (PDB: 1R44) and W155 in VanXYc (PDB: 4OAK), not shown in the figure. These latter are a D-Ala-D-Ala dipeptidase and two D,D-dipeptidases/D,D-carboxypeptidases that mediate transposon-based vancomycin resistance in *Enterococci*.

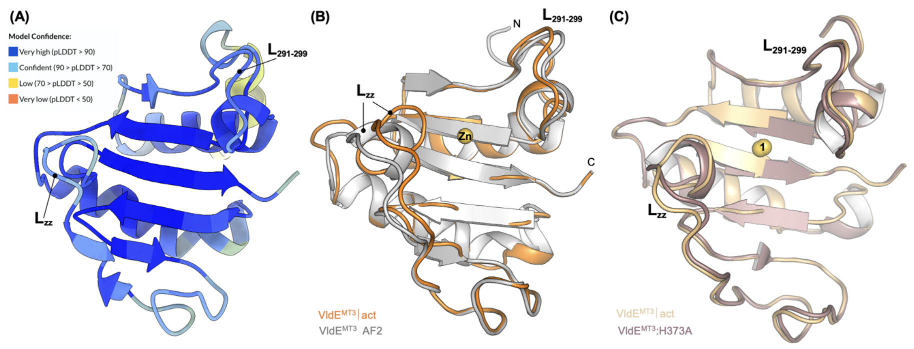

Figure S18. (A) AlphaFold2 predicted structure of VldE^{MT3} domain (UniProt ID: Q8DN78), color-coded according to the model confidence (predicted local distance difference test, pLDDT). **(B)** Superimposition between the VldE^{MT3}:act structure, displayed in orange cartoon, and the structure predicted by AlphaFold2, in gray. The catalytic zinc is represented as a yellow sphere. **(C)** Crystallographic structure of the inactive VldE^{MT3} H373A. Overall superimposition of VldE^{MT3}|act and VldE^{MT3}:H373A structures, depicted in orange and red cartoon respectively.

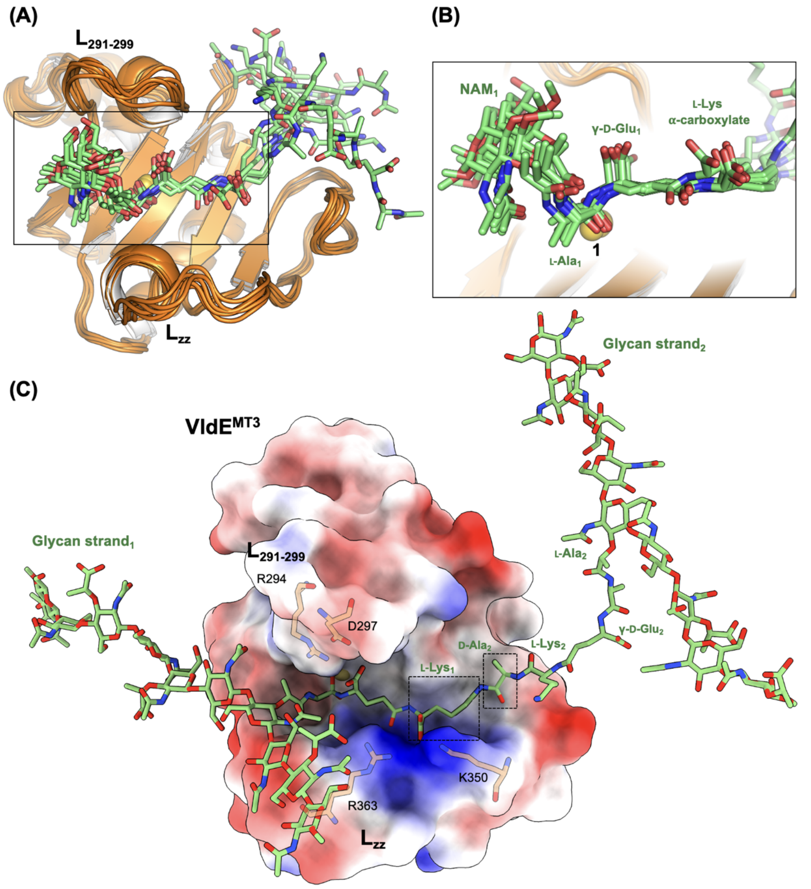

Figure S19. Peptidoglycan recognition by VldE^{MT3}. (A) Structural alignment of modeled complex structures at various time points in the course of dynamics simulation (5, 50, 100, 150, 200, 250, and 300 ns) reveals a stable complex. **(B)** Zoom-in view of the transition-state species for the reaction bound within the active site. **(C)** Computational model of two peptide-linked glycan strands (colored by atom types with green capped sticks for carbons) showing the transition-state species for the reaction bound within the active site of VldE^{MT3}, showed in a electrostatic surface potential representation. Negative charges are shown in red and positive charges are shown in blue. Key residues from VldE^{MT3} predicted to stabilize the carboxylate groups of the substrate are represented as capped sticks.

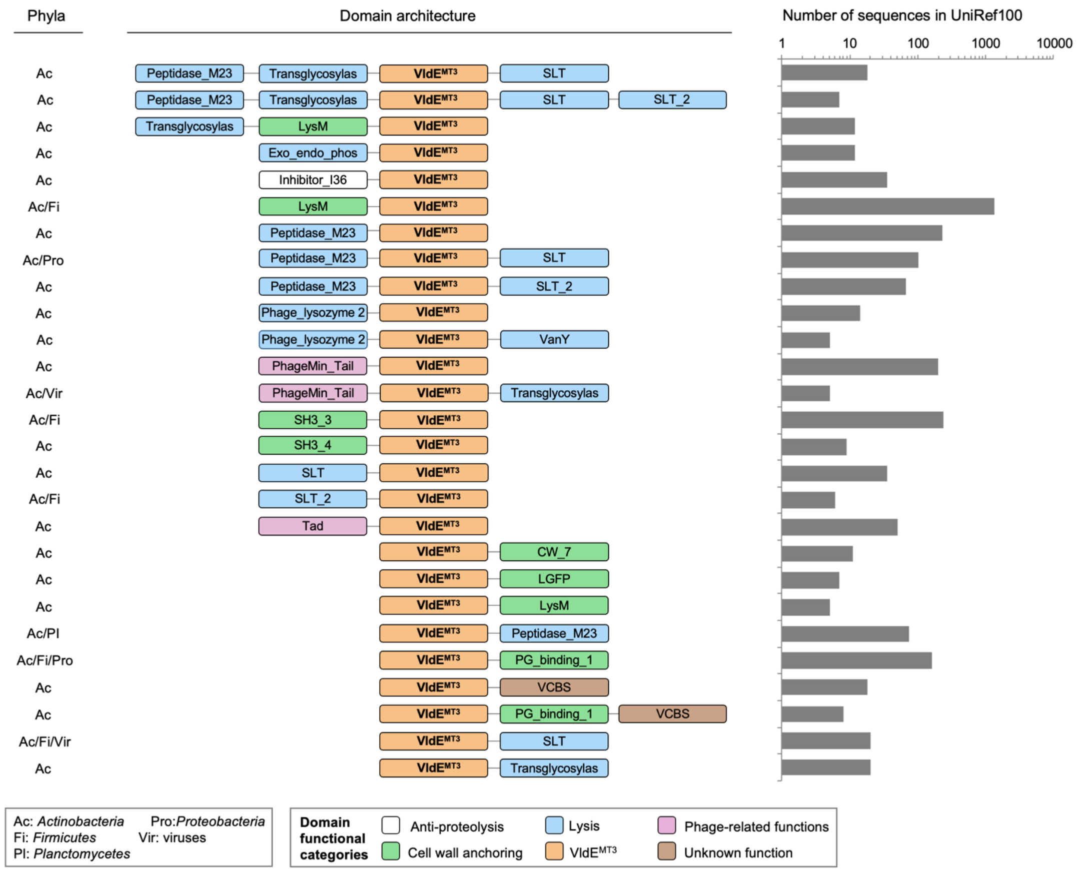

Figure S20. Distribution and domain composition of VldE^{MT3} homologs.

Supplementary Movies

Movie S1- Self-inhibited structure of $VldE^{MT3}$:1Zn displaying an occluded active site after 100 ns of MD simulations.

Movie S2- Representative molecular dynamics trajectory of the apo form of VldE.

Movie S3- Representative molecular dynamics trajectory of the complex between VldE and the transition state of a minimalist representation of the substrate.

Movie S4- Representative molecular dynamics trajectory of the complex between VldE and the transition state of a reduced representation of substrate **8**.

Supplementary Tables

Table S1. Strains and plasmids used in the present study.

Strains and plasmids	Relevant characteristics	Source
E. coli		
DH5a	Plasmid host strain	Invitrogen
BL21 (DE3)	Expression host	Invitrogen
S. pneumoniae		
RH14	R6, Δ*lytA::kan;* Kan^r	Eldholm *et al.*[9]
RH426	R6, contains the janus cassette	Johnsborg *et al.*[10]
RH425	R6, janus compatible	Johnsborg *et al.*[10]
gs551	R6, Δ*spr1875*::janus; Kan^r	This study
Pen6	Penicillin resistant R6 derivate	Zighelboim *et al.*[12]
ds1079	Pen6 but Δ*spr1875*::janus; Kan^r	This study
Spn 5	R800 *rpsL1*	This study
Spn 2211	R800 *rpsL1, Δspr1875::kan-rpsL*	This study
Spn 2948	R800 *rpsL1, spr1875-sfGF*P	This study
Spn 2217	R800 *rpsL1, Δspr1875*	This study
**Plasmids**		
pGS01	Contains the CHiC-encoding sequence	Stamsås *et al.*[13]
pRSET A	Carrying the lacT7 promoter; Amp^r	Invitrogen
pRSET- CHiC-spr1875	*CHiC-spr1875* cloned downstream of P_{lacT7}	This study
pRSET- CHiC-MT3	*CHiC-spr1875*^{MT3} cloned downstream of P_{lacT7}	This study
pRSET- CHiC-MT3(H301A)	*CHiC-spr1875*$^{MT3(H301A)}$ cloned downstream of P_{lacT7}	This study

pRSET- CHiC-MT3(D308A)	*CHiC-spr1875*$^{MT3(D308A)}$ cloned downstream of P_{lacT7}	This study
pRSET- CHiC-MT3(E368A)	*CHiC-spr1875*$^{MT3(E368A)}$ cloned downstream of P_{lacT7}	This study
pRSET- CHiC-MT3(H370A)	*CHiC-spr1875*$^{MT3(H370A)}$ cloned downstream of P_{lacT7}	This study
pRSET- CHiC-MT3(D372A)	*CHiC-spr1875*$^{MT3(H372A)}$ cloned downstream of P_{lacT7}	This study
pRSET- CHiC-MT3(H373A)	*CHiC-spr1875*$^{MT3(H373A)}$ cloned downstream of P_{lacT7}	This study
pRSET- CHiC-MT3(H375A)	*CHiC-spr1875*$^{MT3(H375A)}$ cloned downstream of P_{lacT7}	This study

Table S2. Oligos used for conventional PCR and overlap extension PCR.

Name	Sequence 5'→3'	Restriction site	Source
spr1875			
3371 (Forward primer to amplify upstream region of *spr1875* gene)	CAAGTATTCGGTGCAAGATT		This study
3370 (Reverse primer to amplify downstream region of *spr1875* gene)	TGCTCCAAAGCAACGATCG		This study
**Spn 2211**			
3368	TATCCATTAAAAATCAAACGGTCTTTCA AATTCCTTTCAAA		This study
3379	ATCCATTAAAAATCAAACGGATCTCAT CCTCCTAGAAATA		This study
**Janus Kan-rpsL cassette amplification**			
536	CCGTTTGATTTTTAATGGATAATG		This study
537	AGAGACCTGGGCCCCTTTCC		This study
**Spn 2217**			
3420	ATGATGTTACCAAGTCGGGTTCTTTCAA ATTCCTTTCAAA		This study
3419	TTTGAAAGGAATTTGAAAGAACCCGAC TTGGTAACATCAT		This study
**Spn 2948**			
3754	CTGCAGGAACTCGATGTCTAGTTTTCCA TTCATTGAAACGTGAA		This study
3755	TGGATGAATTGTACAAATAAACCCGA CTTGGTAACATCAT		This study
**Δ*spr1875*::janus**			
Kan484.F	GTTTGATTTTTAATGGATAATGTG		Johnsborg
RpsL41.R	CTTTCCTTATGCTTTTGGAC		Johnsborg[14]
gs67 (1000 bp up)	CAGTCTGGCAGGGATTAGG		This study
gs68 (1000 bp down)	GAAAGACTCCTAGCAGAGC		This study
gs69	CACATTATCCATTAAAAATCAAACTC TTTCAAATTCCTTTCAAATGA		This study

gs70	CTAAACGTCCAAAAGCATAAGGAAA GACCCGACTTGGTAACATCATT		This study
CHiC-spr1875			
ds58 (CHiC rev)	ACCTTGGAAGTACAGGTTCTC		Stamsås *et al.*[13]
ds317 (CHiC fwd)	tacgcatATGCATCATCATCATCATCATg	NdeI	This study
ds163	GAGAACCTGTACTTCCAAGGTGAAGA AGTTCTTTGGACTGCAC		This study
ds165	CGTAGAATTCTTATCCATTCATTGAA ACGTGAAC	EcoRI	This study
**CHiC-Spr1875^{MT3} (*CHiC* fragment amplified using primers ds317 and ds58. Primer ds248 used in combination with ds165 to amplify Spr1875^{MT3})**			
ds248	GAGAACCTGTACTTCCAAGGTGCTGC GCCCGATTATGCTG		This study
**Primers used to introduce point mutations (alanine codon in red) in *spr1875^{MT3}* (used in combination with ds317/ds165 and a *CHiC-spr1875^{MT3}* template for subsequent overlap PCR).**			
ds659 (H301A)	GCAGGAAAAGGTTTGGCTATCGATTT TATG		This study
ds660 (H301A)	CATAAAATCGATAGCCAAACCTTTTC CTGCATCTCCACTGTCTCCTGGAC		This study
ds661 (D308A)	GCATTTATGGTACCAGAACGTTCAG		This study
ds662 (D308A)	CTGAACGTTCTGGTACCATAAATGCG ATAGCCAAACCTTTTCCGTG		This study
ds663 (H370A)	GCATATGATCACGTTCACGTTTCAAT G		This study
ds664 (H370A)	CATTGAAACGTGAACGTGATCATATG CATTTTCTGTCACACTACCACG		This study
ds665 (D372A)	GCACACGTTCACGTTTCAATGAATG		This study
ds666 (D372A)	CATTCATTGAAACGTGAACGTGTGCA TAGTGATTTTCTGTCACACTAC		This study
ds667 (H373A)	GCAGTTCACGTTTCAATGAATGGATA AAC		This study

ds668 (H373A)	GTTTATCCATTCATTGAAACGTGAAC TGCATCATAGTGATTTTCTGTCACAC		This study
ds669 (H375A)	GCAGTTTCAATGAATGGATAAACCC		This study
ds670 (H375A)	GGGTTTATCCATTCATTGAAACTGCA ACGTGATCATAGTGATTTTCTG		This study
ds674 (E368A)	GCAAATCACTATGATCACGTTCACG		This study
ds675 (E368A)	CGTGAACGTGATCATAGTGATTTGCT GTCACACTACCACGGTCTG		This study

Table S3. Data collection and refinement statistics for VldE^{MT3} structures.

	**VldE^{MT3}:4Zn**	**VldE^{MT3}:3Zn**	**VldE^{MT3}:2Zn**	**VldE^{MT3}:1Zn**	**VldE^{MT3}|act**	**VldE^{MT3} H373A**
**Data collection**						
Wavelength (Å)	1.28223	1.28366	1.28218	1.28366	0.979	0.979
Resolution range (Å)	49.25 – 1.50 (1.53 – 1.50)	49.53 – 1.60 (1.63 – 1.60)	51.21 – 1.85 (1.89 – 1.85)	32.40 – 2.80 (2.95 – 2.80)	28.56 – 1.50 (1-53 – 1.50)	29.57 – 1.14 (1.16 – 1.14)
Space group	P3	P3	P3	P3	P3	P3
Unit-cell parameters						
a, b, c (Å)	56.86, 56.86, 32.33	57.19, 57.19, 32.51	59.12, 59.12, 32.68	57.18, 57.18, 32.40	57.11, 57.11, 32.67	59.14, 59.14, 32.48
α, β, γ (°)	90, 90, 120	90, 90, 120	90, 90, 120	90, 90, 120	90, 90, 120	90, 90, 120
No. of observations	184478	139723	100213	18845	193468	456830
Unique reflections	18747 (956)	15685 (753)	10935 (684)	2934 (444)	18946 (921)	46365 (2322)
Redundancy	9.8 (9.5)	8.9 (9.8)	9.7 (10)	6.4 (6.4)	10.2 (10.2)	9.9 (9.5)
CC1/2	0.998 (0.809)	0.990 (0.381)	0.991 (0.342)	0.971 (0.404)	0.986 (0.715)	0.999 (0.401)
Mean I/σ(I)	24.6 (1.6)	7.4 (0.8)	8.9 (1.3)	6.4 (1.7)	6.1 (1.3)	18.8 (1.1)
Wilson B-factor (Å2)	25.5	26.8	31.7	68.4		18.5
Twinning fraction	---	0.284 for -h, -k, l	---	0.428 for -h, -k, l	0.439 for -h, -k, l	0.213 for -h, -k, l
Completeness (%)						
Spherical	100 (100)	99.8 (100)	94.8 (100)	99.8 (99.8)	99.3 (99.1)	100 (100)
Ellipsoidal	---	---	---	---	---	---
**Refinement**						
Resolution range (Å)	32.35 – 1.50 (1.54 – 1.50)	28.60 – 1.60 (1.64 – 1.60)	32.68 – 1.85 (1.90 – 1.85)	14.71 – 2.80 (2.95– 2.80)	28.56 – 1.50 (1.53 – 1.50)	29.57 – 1.14 (1.17 – 1.14)
Rwork/Rfree	0.1957/0.2424	0.2177/0.2539	0.2132/0.2578	0.2370/0.2800	0.1655/0.1987	0.1074/0.1278
Number of atoms	937	944	1766	1680	1054	1933
Protein	856	859	1662	1671	917	1794
Water	67	76	100	0	133	136
Zinc	5	4	3	1	4	3
Cadmium	1	1	1	1	0	0
Acetate	8	4	0	7	0	0
Sulfate	0	0	0	0	0	0
Average B-factors (Å2)	34.0	33.0	37.0	64.0	34.0	21.0
RMSD						
Bond lengths (Å)	0.030	0.010	0.021	0.005	0.003	0.003
Bond angles (°)	1.666	1.760	1.672	0.687	1.008	0.979
Ramachandran favored (%)	96.04	93.20	98.11	88.68	96.16	97.03
Ramachandran allowed (%)	0.00	0.97	0.00	0.00	0.00	0.00
PDB entry	9FLH	9FLJ	9FLK	9FLL	9FLM	9FLN

References:

(1) Arolas, J. L.; Goulas, T.; Cuppari, A.; Gomis-Rüth, F. X. Multiple Architectures and Mechanisms of Latency in Metallopeptidase Zymogens. *Chem Rev* **2018**, *118* (11), 5581–5597.

(2) Piuri, M.; Hatfull, G. F. A Peptidoglycan Hydrolase Motif within the Mycobacteriophage TM4 Tape Measure Protein Promotes Efficient Infection of Stationary Phase Cells. *Mol Microbiol* **2006**, *62* (6), 1569–1585. https://doi.org/10.1111/j.1365-2958.2006.05473.x.

(3) Charlier, P.; Wery, J.-P.; Dideberg, O.; Frère, J.-M. Streptomyces Albus G D -Ala- D -Ala Carboxypeptidase. In *Encyclopedia of Inorganic and Bioinorganic Chemistry*; 2011; pp 1–13. https://doi.org/10.1002/9781119951438.eibc0498.

(4) Hong, S.; Son, B.; Ryu, S.; Ha, N. C. Crystal Structure of LysB4, an Endolysin from Bacillus Cereus-Targeting Bacteriophage B4. *Mol Cells* **2019**, *42* (1), 79–86. https://doi.org/10.14348/molcells.2018.0379.

(5) Prokhorov, D. A.; Mikoulinskaia, G. V.; Molochkov, N. V.; Uversky, V. N.; Kutyshenko, V. P. High-Resolution NMR Structure of a Zn2+-Containing Form of the Bacteriophage T5 L-Alanyl-D-Glutamate Peptidase. *RSC Adv* **2015**, *5* (51), 41041–41049. https://doi.org/10.1039/c5ra05993c.

(6) Odintsov, S. G.; Sabala, I.; Marcyjaniak, M.; Bochtler, M. Latent LytM at 1.3 Å Resolution. *J Mol Biol* **2004**, *335* (3), 775–785. https://doi.org/10.1016/j.jmb.2003.11.009.

(7) Jung-Ho Shin; Alan G. Sulpizio; Aaron Kelley; Laura Alvarez; Shannon G. Murphy; Lixin Fan; Felipe Cava; Yuxin Mao; Mark A. Saper; Tobias Dör. Structural Basis of Peptidoglycan Endopeptidase Regulation. *PNAS* **2020**, *117* (21), 11692–11702. https://doi.org/10.1073/pnas.2001661117/-/DCSupplemental.

(8) Meziane-Cherif, D.; Stogios, P. J.; Evdokimova, E.; Savchenko, A.; Courvalin, P. Structural Basis for the Evolution of Vancomycin Resistance D,D-Peptidases. *Proc Natl Acad Sci U S A* **2014**, *111* (16), 5872–5877. https://doi.org/10.1073/pnas.1402259111.

(9) Eldholm, V.; Johnsborg, O.; Haugen, K.; Ohnstad, H. S.; Havastein, L. S. Fratricide in Streptococcus Pneumoniae: Contributions and Role of the Cell Wall Hydrolases CbpD, LytA and LytC. *Microbiology (N Y)* **2009**, *155* (7), 2223–2234. https://doi.org/10.1099/mic.0.026328-0.

(10) Johnsborg, O.; Håvarstein, L. S. Pneumococcal LytR, a Protein from the LytR-CpsA-Psr Family, Is Essential for Normal Septum Formation in Streptococcus Pneumoniae. *J Bacteriol* **2009**, *191* (18), 5859–5864. https://doi.org/10.1128/JB.00724-09.

(11) Stamsås, G. A.; Straume, D.; Ruud Winther, A.; Kjos, M.; Frantzen, C. A.; Håvarstein, L. S. Identification of EloR (Spr1851) as a Regulator of Cell Elongation in Streptococcus Pneumoniae. *Mol Microbiol* **2017**, *105* (6), 954–967. https://doi.org/10.1111/mmi.13748.

(12) Zighelboim, S.; Tomasz, A. Penicillin-Binding Proteins of Multiply Antibiotic-Resistant South African Strains of Streptococcus Pneumoniae. *Antimicrob Agents Chemother* **1980**, *17* (3), 434–442.

(13) Stamsås, G. A.; Håvarstein, L. S.; Straume, D. CHiC, a New Tandem Affinity Tag for the Protein Purification Toolbox. *J Microbiol Methods* **2013**, *92* (1), 59–63. https://doi.org/10.1016/j.mimet.2012.11.003.

(14) Johnsborg, O.; Eldholm, V.; Bjørnstad, M. L.; Håvarstein, L. S. A Predatory Mechanism Dramatically Increases the Efficiency of Lateral Gene Transfer in Streptococcus Pneumoniae and Related Commensal Species. *Mol Microbiol* **2008**, *69* (1), 245–253. https://doi.org/10.1111/j.1365-2958.2008.06288.x.

(15) Winther, A. R.; Kjos, M.; Stamsås, G. A.; Håvarstein, L. S.; Straume, D. Prevention of EloR/KhpA Heterodimerization by Introduction of Site-Specific Amino Acid Substitutions Renders the Essential Elongasome Protein PBP2b Redundant in Streptococcus Pneumoniae. *Sci Rep* **2019**, *9* (1), 3681. https://doi.org/10.1038/s41598-018-38386-6.
